# Supplementary material for: Postnatal checks and primary care consultations in the year following childbirth: an observational cohort study of 309 573 women in the UK, 2006–2016
Source: BMJ Open. 2020 Nov 23;10(11):e036835. doi: 10.1136/bmjopen-2020-036835 (PMC7684667; doi:10.1136/bmjopen-2020-036835)
Supplement: Supplementary data [file bmjopen-2020-036835supp001.pdf]

Holly Smith, 29/06/20
Postnatal_check_supplementary

Supplementary materials

Likelihood of having a postnatal check using a more sensitive outcome definition (any consultation between weeks 5-10)

Characteristic	All women n	Consultation in weeks 5-10 n (%)	Consultation in weeks 5-10 Unadjusted: IRR (95% CI)	Consultation in weeks 5-10 Age & deprivation adjusted: IRR (95% CI)
**Overall**	309,573	243,516 (78.7)		
**Maternal age (years)**				
15-19	9,568	6,977 (72.9)	0.94 (0.91-0.96)	0.94 (0.92-0.97)
20-24	43,116	32,429 (75.2)	0.96 (0.95-0.98)	0.97 (0.95-0.98)
25-29	77,698	60,597 (78.0)	0.99 (0.98-1.00)	0.99 (0.98-1.00)
30-34	98,269	78,671 (80.1)	1	1
35-39	64,171	51,504 (80.3)	1.00 (0.98-1.01)	1.00 (0.98-1.01)
40-44	15,908	12,674 (79.7)	0.99 (0.97-1.01)	0.99 (0.97-1.01)
45-49	843	664 (78.8)	0.98 (0.90-1.06)	0.97 (0.90-1.06)
**Townsend Score quintile**				
1-least deprived	58,583	48,142 (82.2)	1	1
2	53,656	43,336 (80.8)	1.00 (0.98-1.01)	1.00 (0.98-1.01)
3	62,023	49,169 (79.3)	0.99 (0.97-1.00)	0.99 (0.98-1.00)
4	58,506	45,574 (77.9)	0.98 (0.96-0.99)	0.98 (0.97-1.00)
5-most deprived	44,346	32,729 (73.8)	0.95 (0.93-0.96)	0.95 (0.94-0.97)
Missing	32,459	24,566 (75.7)	Excluded	Excluded
**Mode of delivery**				
Vaginal delivery	75,506	63,533 (86.8)	1	1
Caesarean	23,426	20,074 (85.7)	1.03 (1.01-1.04)	1.02 (1.01-1.04)
Unknown	210,641	159,533 (75.7)	0.96 (0.94-0.98)	0.96 (0.94-0.97)
**Parity**				
First	149,639	118,998 (79.5)	1	1
Second	69,355	53,969 (77.8)	0.97 (0.96-0.98)	0.97 (0.96-0.98)
Third or higher	20,113	15,258 (75.9)	0.95 (0.93-0.96)	0.94 (0.92-0.95)
Unknown	70,466	55,291 (78.5)	0.96 (0.95-0.97)	0.95 (0.94-0.96)
**Smoking status**				
Current smoker	34,634	27,236 (78.6)	0.99 (0.98-1.01)	1.01 (0.99-1.02)
Past smoker**	85,592	66,542 (77.7)	0.97 (0.96-0.98)	0.97 (0.96-0.98)
Non-smoker	143,349	115,019 (80.2)	1	1
Unknown	45,998	34,719 (75.5)	0.93 (0.92-0.94)	0.93 (0.92-0.94)
**Year group**				
2006-2007	63,793	50,496 (79.2)	1	1
2008-2009	66,319	52,571 (79.3)	1.00 (0.98-1.01)	1.00 (0.98-1.01)
2010-2011	66,478	52,819 (79.5)	1.00 (0.99-1.02)	1.01 (0.99-1.02)
2012-2013	63,180	49,788 (78.8)	1.00 (0.99-1.02)	1.00 (0.99-1.02)
2014-2015	49,803	37,842 (76.0)	0.97 (0.96-0.99)	0.97 (0.96-0.99)

* Abbreviations: IRR – incidence rate ratio, CI – confidence interval.
**Practice and woman are included as random effects terms in all models
***Models exclude women with missing Townsend score

Smith HC, *et al. BMJ Open* 2020; 10:e036835. doi: 10.1136/bmjopen-2020-036835

Holly Smith, 29/06/20
Postnatal_check_supplementary

Number of women who had a consultation in weeks 0-4 and/or weeks 5-10, % of cohort

Consultation in week 0-4 (down)/ Consultation in weeks 5-10 (across)	Yes	No	Total
Yes	89,605 (28.9%)	18,723 (6.0%)	108,328 (35.0%)
No	153,911 (49.7%)	47,334 (15.3%)	201,245 (65.0%)
Total	243,516 (78.7%)	66,057 (21.3%)	309,573

Smith HC, *et al. BMJ Open* 2020; 10:e036835. doi: 10.1136/bmjopen-2020-036835
