# Supplementary material for: Postnatal checks and primary care consultations in the year following childbirth: an observational cohort study of 309 573 women in the UK, 2006–2016
Source: BMJ Open. 2020 Nov 23;10(11):e036835. doi: 10.1136/bmjopen-2020-036835 (PMC7684667; doi:10.1136/bmjopen-2020-036835)
Supplement: Author's manuscript [file bmjopen-2020-036835.draft_revisions.pdf]

BMJ Open is committed to open peer review. As part of this commitment we make the peer review history of every article we publish publicly available.

When an article is published we post the peer reviewers' comments and the authors' responses online. We also post the versions of the paper that were used during peer review. These are the versions that the peer review comments apply to.

The versions of the paper that follow are the versions that were submitted during the peer review process. They are not the versions of record or the final published versions. They should not be cited or distributed as the published version of this manuscript.

BMJ Open is an open access journal and the full, final, typeset and author-corrected version of record of the manuscript is available on our site with no access controls, subscription charges or pay-per-view fees (http://bmjopen.bmj.com).

If you have any questions on BMJ Open's open peer review process please email info.bmjopen@bmj.com

BMJ Open

Postnatal checks and primary care consultations in the year following childbirth: an observational cohort study of 309,573 women in the UK, 2006-2016

Journal:	*BMJ Open*
Manuscript ID	bmjopen-2020-036835
Article Type:	Original research
Date Submitted by the Author:	06-Jan-2020

Complete List of Authors:	Smith, Holly Christina; University College London, Primary Care and Population Health<br>Saxena, Sonia; Imperial College London, School of Public Health;<br>Petersen, Irene; University College London Medical School, Department of Primary Care and Population health

Keywords:	Maternal medicine < OBSTETRICS, PRIMARY CARE, EPIDEMIOLOGY, REPRODUCTIVE MEDICINE

SCHOLARONE™
Manuscripts

I, the Submitting Author has the right to grant and does grant on behalf of all authors of the Work (as defined in the below author licence), an exclusive licence and/or a non-exclusive licence for contributions from authors who are: i) UK Crown employees; ii) where BMJ has agreed a CC-BY licence shall apply, and/or iii) in accordance with the terms applicable for US Federal Government officers or employees acting as part of their official duties; on a worldwide, perpetual, irrevocable, royalty-free basis to BMJ Publishing Group Ltd ("BMJ") its licensees and where the relevant Journal is co-owned by BMJ to the co-owners of the Journal, to publish the Work in this journal and any other BMJ products and to exploit all rights, as set out in our licence.

The Submitting Author accepts and understands that any supply made under these terms is made by BMJ to the Submitting Author unless you are acting as an employee on behalf of your employer or a postgraduate student of an affiliated institution which is paying any applicable article publishing charge ("APC") for Open Access articles. Where the Submitting Author wishes to make the Work available on an Open Access basis (and intends to pay the relevant APC), the terms of reuse of such Open Access shall be governed by a Creative Commons licence – details of these licences and which Creative Commons licence will apply to this Work are set out in our licence referred to above.

Other than as permitted in any relevant BMJ Author's Self Archiving Policies, I confirm this Work has not been accepted for publication elsewhere, is not being considered for publication elsewhere and does not duplicate material already published. I confirm all authors consent to publication of this Work and authorise the granting of this licence.

For peer review only

Holly Smith, 06/01/20
Postnatal_check_manuscript

Title:

Postnatal checks and primary care consultations in the year following childbirth: an observational cohort study of 309,573 women in the UK, 2006-2016

Authors:

Holly Christina Smith (0000-0001-6805-680X), Sonia Saxena (0000-0003-3787-2083), Irene Petersen (0000-0002-0037-7524).

Holly Christina Smith, PhD student, Department of Primary Care and Population Health, University College London, Royal Free Campus, Rowland Hill Street, London, NW3 2PF.

Sonia Saxena, Professor of Primary Care, School of Public Health, Faculty of Medicine, Imperial College London, 332 Reynolds Building, Charing Cross Campus, W6 8RP.

Irene Petersen, Professor of Epidemiology and Health Informatics, Department of Primary Care and Population Health, Institute of Epidemiology & Health, University College London, Royal Free Campus, Rowland Hill Street, London, NW3 2PF.

Correspondence to: Holly Christina Smith**:** Holly.dorning.18@ucl.ac.uk.

Word count:

3,134

For peer review only

For peer review only - http://bmjopen.bmj.com/site/about/guidelines.xhtml

Abstract

Objective: To describe women's uptake of postnatal checks and primary care consultations in the year following childbirth.

Design: Observational cohort study using electronic health records.

Setting: UK primary care.

Participants: Women aged 16-49 years who had a single live birth recorded in The Health Improvement Network (THIN) primary care database between 2006 and 2016.

Main outcome measures: Postnatal checks and direct consultations in primary care in the year following childbirth.

Results: We examined 1,427,710 consultations in 309,573 women who gave birth to 241,662 children between 2006 and 2016. Of these women, 135,512 (43.8%) had no record of a postnatal check. Teenage women (aged 16 to 19 years) were 12% less likely to have a postnatal check compared with those aged 30-35 years (incidence rate ratio (IRR)=0.88, 95% CI: 0.85-0.91) and those living in the most deprived vs. least areas were 10% less likely to have a postnatal check (IRR=0.90, 95% CI: 0.88-0.92). Women consulted on average 4.8 times per women per year and 293,049 women (94.7%) had at least one direct consultation in the year after childbirth. Consultation rates were higher for smokers (5.9 per women per year, 95% CI: 5.9-5.9) and those with a caesarean delivery (7.7 per women per year, 95% CI: 7.7-7.8). Consultation rates peaked during weeks 5-10 following birth (11.8 consultations/ 100 women) coinciding with the postnatal check; after week ten this fell to 1 consultation/ 100 women.

Conclusions: Four in ten women have no record of receiving a postnatal check within the first ten weeks after giving birth. Teenagers and those from the most deprived areas are among the least likely to have a check. We estimate up to 350,400 women per year in the UK may be missing these opportunities for timely health promotion and to have important health needs identified following childbirth.

Keywords: Maternal Health, Postnatal Care, Primary Health Care, Electronic Health Records, Postpartum Period

For peer review only - http://bmjopen.bmj.com/site/about/guidelines.xhtml

Strengths and limitations of this study

- A major strength of this study is that it is among the largest representative population based studies to date of postnatal care in the first 12 months.
- We drew on data from electronic health records which reflect real-world clinical practice in UK primary care.
- We are limited by what is recorded in a woman's electronic health record and genuine postnatal checks may have occurred in reality which were not accurately coded in primary care data.
- This study may underestimate the number of women who had a postnatal check compared with estimates based on survey data.

For peer review only

Introduction

Providing high quality comprehensive postnatal primary care is a global goal for improving maternal and child health in the first year of life [1]. In the United Kingdom (UK), nearly 800,000 women give birth each year [2] and every woman has access to midwives and health visitors for the first few days after delivery. They are then discharged to the care of their General Practitioner (GP) who invites them for a planned postnatal check 6-8 weeks after birth [3], as recommended by National Institute for Health and Care Excellence (NICE)[4] and the World Health Organisation (WHO) as part of routine postnatal care[5]. The maternal postnatal check provides a unique and timely opportunity for new mothers and health care professionals to evaluate their physical and mental the health mental and physical, assess how women are recovering after pregnancy and birth[6]. The postnatal check is also a point where women and primary care health professionals can discuss breastfeeding, postpartum contraception, smoking cessation, return to physical activity, and dietary advice, particularly after gestational weight gain. GPs also play a role in supporting parents to cope with managing day to day care and minor illness of infants and identify safeguarding concerns for new mothers and their babies.

The postnatal period is typically defined as the first six weeks after childbirth [7] and previous studies estimate 47% to 83% of women will report at least one health problem around eight weeks postpartum [8-10]. Historically, it was anticipated women would recover from pregnancy within this time [11]; however, there is increasing evidence that women have ongoing health needs throughout the first year [12] and even longer [13 14]. For example, up to 5% of women will require ongoing management of medical complications of gestational diabetes[15]; and others may need support with postpartum conditions such as postnatal depression which occurs in 1 in 6 [16]. Current research may underestimate maternal morbidity as many women do not report symptoms or may be reluctant to seek support after childbirth [17].

From previous cross-sectional surveys from 1995 and 2010-2014, it would appear that up to 90% of women attend their postnatal checks, but selection bias of survey participants may overestimate this figure [18-20]. We found no contemporary studies showing patterns of preventive and responsive postnatal care for women. The aim of this observational cohort study was to examine the prevalence of postnatal checks and primary care consultations for women in the first year after childbirth.

For peer review only - http://bmjopen.bmj.com/site/about/guidelines.xhtml

Methods

UK healthcare

In the UK, healthcare is free at the point of delivery for all residents as part of the National Health Service (NHS). Primary care is typically the first point of contact and is largely delivered by GPs and other health care professionals (nurses and health visitors) within a practice. Information about patients and their health are collected during primary care consultations. This information is primarily used for clinical care but is also widely used for research through large healthcare databases.

Data source

We used one of the largest UK primary care databases, The Health Improvement Network (THIN) database. As of December 2016, THIN contained anonymised electronic health records for 16 million registered patients from 730 practices across the UK [21]. The database contains patient-level information on: demographics, prescribing, symptoms, procedures, prevention, lifestyle factors and diagnostics. Consultations can be linked to give a comprehensive picture of someone's care. Diagnostic and symptomatic information are categorised using Read codes, a hierarchical coding system [22]. Additional Health Data codes classify prevention and lifestyle information. Socioeconomic information is captured through Townsend score which provides a measure of material deprivation based on: where a person lives, unemployment, car ownership, home ownership and household overcrowding [23]. THIN is broadly representative of the UK population in terms of demographics, chronic disease and mortality; however, more people live in more affluent areas compared to the general population [24].

Study population

A cohort of women who have given single live birth has previously been identified within THIN [25]. Births and date of childbirth were identified using a combination of an antenatal record, delivery record, postnatal care record, date of last menstrual period, or birth of a child matched to mother's record. To remove more complex births, multiple deliveries (twins, triplets etc.) were excluded, as well as known miscarriage, termination or stillbirths. This cohort contains information on approximately 650,000 pregnancies/childbirths in 1990-2016. In this study, we included women aged 15 to 49 years who gave birth between 1st January 2006 and 31st December 2015 from this cohort. Data quality criteria were also applied whereby practices which did not have acceptable computer use (ACU) or acceptable mortality rates (AMR) by the date of childbirth were excluded.

For peer review only

For peer review only - http://bmjopen.bmj.com/site/about/guidelines.xhtml

ACU is the date a practice was continuously entering on average at least two therapy records, one medical record and one additional health data record per patient per year [26]; and AMR is the date a practice has comparable mortality rates to the rest of the UK, given the size and demographics of the practice [27]. Women who had been registered at a practice for less than six months were also excluded. It was possible for women to have multiple childbirths in this study. Women were followed-up for 12 months to identify their primary use after each childbirth, censoring for maternal death or practice transfer.

Definition of variables

Postnatal check

We identified a postnatal check as any consultation at the time of the check (typically weeks 6-8) which had a specific Read code (beginning with '62R' or '62S') or Additional Health Data code ('1044100000' or '1044000000') identifying it as postnatal visit and/or check. Some women may receive this check slightly earlier or later, and on reviewing the data this window was expanded to weeks 5-10 after birth to include a peak in consultations. We identified substantial variation in the use of these codes by practices and change over time (data not shown). Therefore, we used a second and more sensitive approach where we assumed any consultation at the time of the routine appointment (weeks 5-10) was an opportunity for a postnatal check. The results of this second approach are included in supplementary materials.

Consultations

A primary care consultation was defined as any direct contact between a patient and a healthcare professional taking place: in practice, in a patient's home or by telephone. It was assumed only one consultation took place each day for each woman, therefore multiple records on the same date were grouped.

Patient and childbirth characteristics

We stratified our analysis by maternal age, parity, Townsend score (described previously), smoking status, year (two-year bands) and mode of delivery. Women were assigned to a five-year bands according to their age. We used information in the mother's records as well as children registered within the same household at the time of birth to assign parity (categorised as: First, Second, Third or higher, or Unknown). We used Townsend score quintiles whereby each women is assigned to one of five groups of deprivation, from least to most deprived. We assigned women's smoking status as 'current smoker' (record of smoking at any time in the year after childbirth), 'past smoker' (record of smoking or being an ex-smoker in the two years prior to childbirth and not a current smoker), 'non-

For peer review only

smoker' or 'unknown'. Mode of delivery was determined using the identifying pregnancy/childbirth Read codes and was broadly grouped into 'caesarean', 'vaginal' and 'unknown' based on classifications developed previously [25].

Statistical analysis

A table was derived to show characteristics of women at each childbirth. The crude consultation rate was calculated as the total number of consultations per total person-years, stratified by characteristics. To explore variation across the first year, consultation rate was calculated as the number of consultations on each day with number of women registered with a practice on that day as the denominator. Women who died or transferred practice were censored from the denominator each day. To examine those who had a postnatal check, firstly we calculated the crude proportion of women with the outcome in each patient strata. To explore variation by characteristic in more detail, we examined the likelihood of having a postnatal check between 5-10 weeks in women with at least five weeks follow-up and complete deprivation (Townsend score) information. We developed mixed-effects Poisson models to estimate how the likelihood of having a postnatal check between weeks 5-10 varied by maternal age, Townsend score, mode of delivery, parity, smoking status and year. Three models were developed: unadjusted, age-adjusted and age-deprivation adjusted. Practice and woman (as women can have multiple childbirths) were included as random effects terms, and the log of follow-up time (between weeks 5-10) was included as an offset. All analyses were conducted using Stata V.16 (StataCorp, College Station, Texas, USA).

Patient and public involvement

Two public panels interested in primary care research provided feedback on the study outline. The groups were supportive of the idea to identify attendance of postnatal checks, they suggested we clearly identify groups who do not attend and to examine differences in a woman's first vs subsequent childbirths. As a result, we have included additional analysis exploring attendance by patient characteristics and parity.

For peer review only - http://bmjopen.bmj.com/site/about/guidelines.xhtml

Results

Participants

Between 1 January 2006 and 31 December 2016, 438,538 pregnancies/childbirths were identified in the pregnancy cohort within THIN data. Study inclusion and exclusion criteria were applied to these records which resulted in a final sample of 309,573 childbirths (Figure 1).

Characteristics of women

We identified 309,573 childbirths in this study related to 241,662 women. At childbirth, a third of the women were aged 30-34 years (31.7%). There were 21.1% in the least deprived Townsend quintile compared 16.0% in the most deprived (which is similar to the overall distribution in THIN [24]). Three quarters of women had a vaginal delivery and the rest had a caesarean birth (76.3% vs 23.7%). Of these, nearly half were a first birth (48%) and 22% were a second birth. Half of women were non-smokers (46.3%), compared to 11.2% being current smoker (Table 1).

Postnatal check

Overall, just over half of the women in our study (56%) had a postnatal check, i.e. 44% had no such records (Table 1). In this crude analysis, younger women and those from the most deprived areas were less likely to have a postnatal check (48% of those aged 15-19 years vs 59.5% of those aged 35-39 years; and 47.7% of those from the most deprived area vs 62.7% from the least).

After excluding those with less than five weeks of follow-up information, missing deprivation information, 275,577 women were included in additional analysis (Figure 1). Those aged 15-19 years were 12% less likely (incidence rate ratio (IRR)=0.88, 95% CI: 0.85-0.91) to have a postnatal check between weeks 5-10 relative to women aged 30-35 years (Table 2). Similarly, women from the most deprived areas were 10% less likely (IRR=0.90, 95% CI: 0.88-0.92) to have a postnatal check relative to those from the least deprived areas. Differences across other characteristics were less pronounced; and, adjusting for age, and age and deprivation, had little impact on differences across these other characteristics. The same trend across age and deprivation was identified when using a more sensitive approach to identify a potential postnatal check (any consultation between weeks 5-10), but with a higher proportion of women (78.7%) having a consultation (see supplementary material).

Primary care consultations

For peer review only - http://bmjopen.bmj.com/site/about/guidelines.xhtml

Following the 309,573 childbirths, the majority (94.7%, n=293,049) of women had at least one direct consultation in the year after childbirth. A total of 1,427,710 direct consultations were identified, with women consulting on average 4.8 times/person-year (Table 3). The largest differences in consultation rate compared to the average is seen in those who had a caesarean delivery (7.7/ person-year, 95% CI: 7.7-7.8) and in current smokers (5.9/ person-year, 95% CI: 5.9-5.9). Consultation rates were broadly similar across other characteristics.

Across the first year the consultation rate was highest between weeks 5-10, with a peak of 11.8 consultations/ 100 women in week 6, coinciding with the postnatal check. Following this, the consultation rate fell to an average of 1 consultation/ 100 women (Figure 2).

For peer review only

For peer review only - http://bmjopen.bmj.com/site/about/guidelines.xhtml

Discussion

Main findings

We found that just over half of the women had a record of a postnatal check which means four in ten women (44%) have missed out. Teenage women (aged 15-19 years) were 12% less likely to have a postnatal check compared with older women aged 30-35 years, and those living in the most deprived areas were 10% less likely compared to women from least deprived areas. Women consulted on average 4.8 times per year in the year after childbirth and 94.7% of women had at least one consultation in the year after childbirth. Those who had a caesarean delivery and smokers had higher than average consultation rates (7.7 times per women per year and 5.9 times per women per year respectively). Across the first year, the consultation rate is highest in week six with a peak of 11.8 consultations/ 100 women, which coincides with the postnatal check, after week ten the consultation rate is flat with 1 consultation/ 100 women on each day.

Study strengths and weaknesses

This is among the largest (299,688 person years) representative population based studies to date of postnatal care in the first 12 months. The use of electronic health records provides a reflection of real-world clinical practice allowing us to explore use of the postnatal check in a broad population (not reliant on patient participation). As with all studies of electronic health records however, we are limited by what has been recorded in a woman's record which could mean patient, birth and consultation characteristics may be missing or not accurate. We also recognise the limitations in using Read codes/AHD codes only to identify a postnatal check as there is variation in the use of these by general practice and genuine checks may not be coded as such in primary care data. To account for this, we repeated our analysis using a more sensitive definition of a postnatal check (any consultation in week 5-10). While we found a larger proportion (78.7%) having been in contact with primary care, the overall trends in terms of the sociodemographic information was consistent between the two approaches.

Findings in relation to previous studies

Previous studies estimate that 85% to 91% of women in England have a postnatal check [18 19]; however only 56% of women in our study had evidence of having one. We would expect our estimates to be lower as we used electronic health records which capture a broader picture of real-world practice compared with previous studies that may have been subject to selection and recall bias. When using a more sensitive approach, where we considered any consultation in weeks 5-10 as

For peer review only - http://bmjopen.bmj.com/site/about/guidelines.xhtml

evidence of a postnatal check, our findings are closer to those of previous studies, although we are aware that not all of these consultations would have covered topics meant for the postnatal check. It is likely the true number having a check lies between our two estimates. We also identified that those from more deprived areas were less likely to have a postnatal check which supports previous findings [20]. The consultation rate of 4.8 per person per year identified in our study is comparable to that found by others, when taking age, sex and reason for consultation into account [28].

There are several possible explanations for our findings of a low uptake of postnatal checks. It is possible that women do not want or feel they need advice from GPs; or invitations from the GP are not taken up either because women do not respond to them, or may find it difficult to access appointments. Alternatively a lack of recording in electronic health records may explain the apparently low rate of postnatal checks.

Implications of findings and future research recommendations

It is encouraging to find that the majority of women return to primary care at least once in the year after childbirth; however, it is concerning that four in ten women did not have a structured postnatal check. In UK, approximately 800,000 women give birth each year[2], our estimates (44%) then suggest that up to 350,400 women may be missing this key check. The postnatal period is a potentially vulnerable time for women and there could be serious consequences to not identifying women at risk of poor health or harm after childbirth [29]. The postnatal check has been shown to be a key contact to identify serious health needs such as postnatal depression, which affects 1 in 6 women after childbirth. [25] It also provides protected time and opportunities to improve women's health and wellbeing through preventative intervention such as timely access to contraception, advice about weight management or diet following gestational weight gain, or support to stop smoking can be given [4]. Our finding that younger women and those from more deprived areas are less likely to have a check is particularly important as they may be most likely to benefit. For example, contraceptive uptake is particularly low in younger and more deprived groups [30], and offering timely access through the check could lead to fewer unwanted or repeat pregnancies for these women.

Our findings suggest practices may need to implement systems for follow up of women who have declined or missed a postnatal check. There is a need for better promotion of the benefits of attending the postnatal check at other times in the maternity pathway; such as during midwife or health visitor appointments, in hospital or birth units, or at other GP maternity and baby check-ups. Additionally, there are currently no known financial or quality-based incentives to document primary care activity in the postnatal period. This could lead to variation in services and underreporting of

For peer review only - http://bmjopen.bmj.com/site/about/guidelines.xhtml

activity. It is vital to improve the documentation of this care to more accurately understand women's use of the postnatal check, more broadly their health needs and service use after childbirth, and ultimately improve care. We recommend more research to explore the reasons behind the low uptake of postnatal checks.

Lastly, in this study our focus was on who had a postnatal check, and while NICE outlines the content of these appointments, few studies have explored what health needs are covered in actuality. This should be explored through further research to better understand what content, delivery, timing and frequency of postnatal checks are most effective for women and if attending a postnatal check leads to better outcomes.

Conclusion

Four in ten women have no record of receiving a postnatal check within the first ten weeks after giving birth, this is despite the majority of women returning to primary care at least once in the year after childbirth. Teenagers and those from the most deprived areas are among the least likely to have a check. We estimate up to 350,400 women per year in the UK may be missing these opportunities for timely health promotion and to have important health needs identified after childbirth.

For peer review only

For peer review only - http://bmjopen.bmj.com/site/about/guidelines.xhtml

Acknowledgements

We are very grateful to members of the public who generously gave their time to feedback on the outline of this study, in particular we are grateful to the many women who have shared their experiences of childbirth and beyond, and who serve as a continued source of inspiration for this research. We are also appreciative for the support of our funders.

Footnotes

Authors contributions

This study was designed and conceived by HCS, SS and IP. HCS conducted the data analysis and wrote the first draft of this manuscript. SS and IP made comments on the manuscript. HCS and IP had full access to the database and guarantee this manuscript is an honest, accurate, and transparent account of the study being reported; that no important aspects of the study have been omitted; and that any discrepancies from the study as planned have been explained.

Funding

This study is funded by the National Institute for Health Research (NIHR) School for Primary Care Research (SPCR) (project reference 549832). The views expressed are those of the authors and not necessarily those of the NIHR or the Department of Health and Social Care.

SS is funded by the National Institute for Health Research (NIHR) School for Public Health Research (SPHR) and NIHR Northwest London Applied Research Collaboration (ARC). The School for Public Health Imperial College London is also grateful for support from the Imperial NIHR Biomedical Research Centre.

The funders had no role in study design, data collection and analysis, decision to publish, or preparation of the manuscript.

Data sharing statement

No additional data are available.

Competing interests

All authors have completed the ICMJE uniform disclosure form at www.icmje.org/coi_disclosure.pdf and declare: HCS reports grants from National Institute of Health Research School for Primary Care Research and SS reports grants from NIHR School for Public Health Research, grants from NIHR NW

For peer review only - http://bmjopen.bmj.com/site/about/guidelines.xhtml
For peer review only

London Applied Research Collaboration, during the conduct of the study; grants from The Daily Mile Foundation Charity, outside the submitted work; no other relationships or activities that could appear to have influenced the submitted work.

Ethical approval and data access

Approval was received from the Scientific Review Committee on 10/04/2019 (THIN protocol number: 19THIN013). THIN is a registered trademark of Cegedim SA in the UK and other countries. Reference made to THIN database is intended to be descriptive of the data asset licensed by IQVIA. This work uses de-identified data provided by patients as a part of their routine primary care.

Copyright/license for publication

The Corresponding Author HCS has the right to grant on behalf of all authors and does grant on behalf of all authors, a worldwide licence to the Publishers and its licensees in perpetuity, in all forms, formats and media (whether known now or created in the future), to i) publish, reproduce, distribute, display and store the Contribution, ii) translate the Contribution into other languages, create adaptations, reprints, include within collections and create summaries, extracts and/or, abstracts of the Contribution, iii) create any other derivative work(s) based on the Contribution, iv) to exploit all subsidiary rights in the Contribution, v) the inclusion of electronic links from the Contribution to third party material where-ever it may be located; and, vi) licence any third party to do any or all of the above.

As our data are anonymised it is not possible to disseminate our findings to study participants.

For peer review only

For peer review only - http://bmjopen.bmj.com/site/about/guidelines.xhtml

References

1. United Nations. Transforming our world: The 2030 agenda for sustainable development, 2015.
2. Office for National Statistics. Births by mothers' usual area of residence in the UK [Data file]. Office for National Statistics, London, 2016. [cited 12 Dec 2019]. Avaliable from: https://www.ons.gov.uk/peoplepopulationandcommunity/birthsdeathsandmarriages/livebirths/datasets/birthsbyareaofusualresidenceofmotheruk.
3. NHS Choices. Your 6-week postnatal check [Available from: https://www.nhs.uk/conditions/pregnancy-and-baby/postnatal-check/ accessed 20/12/2018.
4. National Institute for Health and Care Excellence. Postnatal care up to 8 weeks after birth. NICE Clinical Guideline 37, 2006. https://www.nice.org.uk/guidance/cg37.
5. World Health Organisation, United Nations Population Fund, Unicef. Pregnancy, Childbirth, Postpartum and Newborn Care: A guide for essential practice. Third ed. Geneva, 2015.
6. Jakes AD, Oakeshott P, Bick D. The maternal six week postnatal check. *BMJ* 2019;367:l6482. doi: 10.1136/bmj.l6482 [published Online First: 2019/12/04]
7. World Health Organisation. WHO Technical Consultation on Postpartum and Postnatal Care, 2010.
8. Glazener CM, Abdalla M, Stroud P, et al. Postnatal maternal morbidity: extent, causes, prevention and treatment. *Br J Obstet Gynaecol* 1995;102(4):282.
9. Thompson JF, Roberts CL, Currie M, et al. Prevalence and persistance of health problems after childbirth: associations with parity and method of birth. *Birth* 2002;29(2):83-94.
10. MacArthur C, Lewis M, Knox EG. Health after childbirth. *Br J Obstet Gynaecol* 1991;98:1193-204.
11. Albers LL. Health problems after childbirth. *Journal of Midwifery & Women's Health* 2000;45(1):55-57.
12. Ansara D, Cohen MM, Gallop R, et al. Predictors of women's physical health problems after childbirth. *Journal of Psychosomatic Obstetrics & Gynecology* 2009;26(2):115-25. doi: 10.1080/01443610400023064
13. Dahlen HG. Urinary incontinence: where to now? *BJOG* 2016;123(6):1030. doi: 10.1111/1471-0528.13427 [published Online First: 2015/05/27]
14. MacArthur C, Wilson D, Herbison P, et al. Faecal incontinence persisting after childbirth: a 12 year longitudinal study. *BJOG* 2013;120(2):169-79. doi: 10.1111/1471-0528.12039 [published Online First: 2012/11/30]
15. National Institute for Health and Care Excellence. Diabetes in pregnancy: management from preconception to the postnatal period. NICE Guideline No. 3, 2015. https://www.nice.org.uk/guidance/ng3.
16. Shorey S, Chee CYI, Ng ED, et al. Prevalence and incidence of postpartum depression among healthy mothers: A systematic review and meta-analysis. *J Psychiatr Res* 2018;104:235-48. doi: 10.1016/j.jpsychires.2018.08.001 [published Online First: 2018/08/17]
17. Chou D, Tuncalp O, Firoz T, et al. Constructing maternal morbidity - towards a standard tool to measure and monitor maternal health beyond mortality. *BMC Pregnancy Childbirth* 2016;16:45. doi: 10.1186/s12884-015-0789-4 [published Online First: 2016/03/05]
18. Bick DE, MacArthur C. Attendance, content and relevance of the six week postnatal examination. *Midwifery* 1995(11):69-73.
19. Redshaw M, Heikkila K. Delivered with care: a national survey of women's experience of maternity care 2010: National Perinatal Epidemiology Unit, 2010.
20. Redshaw M, Henderson J. Safely delivered: a national survey of women's experiences of maternity care 2014: National Perinatal Epidemiology Unit, 2015.
21. Quintiles IMS. The Health Improvement Network Data Guide for Researchers, 2017.
22. Booth N. What are the Read Codes? *Health Libr Rev* 1994;11:177-82.

For peer review only - http://bmjopen.bmj.com/site/about/guidelines.xhtml

23. Townsend P, Phillimore P, Beattie A. Health and deprivation: inequality and the north. London: Routledge 1989.
24. Blak BT, Thompson M, Dattani H, et al. Generalisability of The Health Improvement Network (THIN) database: demographics, chronic disease prevalence and mortality rates. *Inform Prim Care* 2011;19:251-5.
25. Petersen I, McCrea RL, Sammon CJ, et al. Risks and benefits of psychotropic medication in pregnancy: cohort studies based on UK electronic primary care health records. *Health Technol Assess* 2016;20(23):1-176. doi: 10.3310/hta20230 [published Online First: 2016/04/01]
26. Horsfall L, Walters K, Petersen I. Identifying periods of acceptable computer usage in primary care research databases. *Pharmacoepidemiol Drug Saf* 2013;22(1):64-9. doi: 10.1002/pds.3368 [published Online First: 2012/11/06]
27. Maguire A, Blak BT, Thompson M. The importance of defining periods of complete mortality reporting for research using automated data from primary care. *Pharmacoepidemiol Drug Saf* 2009;18(1):76-83. doi: 10.1002/pds.1688 [published Online First: 2008/12/10]
28. Wang Y, Hunt K, Nazareth I, et al. Do men consult less than women? An analysis of routinely collected UK general practice data. *BMJ Open* 2013;3(8) doi: 10.1136/bmjopen-2013-003320
29. Knight M, Bunch K, Tuffnell D, et al. Saving Lives, Improving Mothers' Care: Lessons learned to inform maternity care from the UK and Ireland Confidential Enquiries into Maternal Deaths and Morbidity 2014–16, 2018.
30. National Institute for Health and Care Excellence. Contraceptive services for under 25s. NICE Public health guideline No. 51, 2014. www.nice.org.uk/guidance/ph51.

For peer review only

For peer review only - http://bmjopen.bmj.com/site/about/guidelines.xhtml

Tables

Table 1: Characteristics of women at childbirth and number with a postnatal check

Characteristic	All women n	Record of postnatal check in weeks 5-10 n (% across the row)
**Overall**	309,573	174,061 (56.2)
**Maternal age (years)**		
15-19	9,568	4,599 (48.1)
20-24	43,116	21,763 (50.5)
25-29	77,698	42,417 (54.6)
30-34	98,269	57,308 (58.3)
35-39	64,171	38,154 (59.5)
40-44	15,908	9,347 (58.8)
45-49	843	473 (56.1)
**Townsend Score quintile**		
1-least deprived	58,583	36,752 (62.7)
2	53,656	32,326 (60.3)
3	62,023	35,413 (57.1)
4	58,506	31,601 (54.0)
5-most deprived	44,346	21,138 (47.7)
Missing	32,459	16,831 (51.9)
**Mode of delivery**		
Vaginal delivery	75,506	46,634 (61.8)
Caesarean	23,426	14,384 (61.4)
Unknown	210,641	113,043 (53.7)
**Parity**		
First	149,639	84,010 (56.1)
Second	69,355	39,269 (56.6)
Third or higher	20,113	10,781 (53.6)
Unknown	70,466	40,001 (56.8)
**Smoking status**		
Current smoker	34,634	18,199 (52.6)
Past smoker	85,592	47,464 (55.5)
Non-smoker	143,349	82,420 (57.5)
Unknown	45,998	25,978 (56.5)
**Year group**		
2006-2007	63,793	36,863 (57.8)
2008-2009	66,319	38,124 (57.5)
2010-2011	66,478	37,897 (57.0)
2012-2013	63,180	34,896 (55.2)
2014-2015	49,803	26,281 (52.8)

For peer review only - http://bmjopen.bmj.com/site/about/guidelines.xhtml

Table 2: Mixed-effects Poisson estimates of the likelihood of having a postnatal check by age, Townsend score, mode of delivery, parity, smoking status and year group; unadjusted, and adjusted for age and deprivation

		Record of postnatal check in weeks 5-10	
**Characteristic**	**n (%)**	Unadjusted: IRR (95% CI)	Age & deprivation adjusted: IRR (95% CI)
**Overall**	275,577		
**Maternal age (years)**			
15-19	8,704 (3.2)	0.88 (0.85-0.91)	0.89 (0.87-0.92)
20-24	38,503 (14.0)	0.92 (0.91-0.94)	0.93 (0.92-0.95)
25-29	68,751 (25.0)	0.97 (0.96-0.98)	0.97 (0.96-0.99)
30-34	86,889 (31.5)	1	1
35-39	57,533 (20.9)	1.00 (0.99-1.02)	1.00 (0.99-1.01)
40-44	14,428 (5.2)	0.99 (0.97-1.01)	0.99 (0.97-1.01)
45-49	769 (0.3)	0.96 (0.87-1.05)	0.96 (0.87-1.05)
**Townsend Score quintile**			
1-least deprived	58,304 (21.2)	1	1
2	53,370 (19.4)	0.99 (0.98-1.01)	1.00 (0.98-1.01)
3	61,681 (22.4)	0.97 (0.95-0.98)	0.97 (0.96-0.99)
4	58,165 (21.1)	0.95 (0.93-0.97)	0.96 (0.95-0.98)
5-most deprived	44,057 (16.0)	0.90 (0.88-0.92)	0.92 (0.90-0.93)
**Mode of delivery**			
Vaginal delivery	68,202 (76.6)	1	1
Caesarean	20,828 (23.4)	1.02 (1.00-1.04)	1.01 (0.99-1.03)
Unknown	186,547	0.99 (0.97-1.01)	0.99 (0.97-1.01)
**Parity**			
First	132,164 (48.0)	1	1
Second	62,535 (22.7)	0.99 (0.98-1.01)	0.98 (0.97-0.99)
Third or higher	18,504 (6.7)	0.95 (0.93-0.97)	0.94 (0.92-0.96)
Unknown	62,374 (22.6)	0.96 (0.95-0.97)	0.95 (0.94-0.96)
**Smoking status**			
Current smoker	31,494 (11.4)	0.94 (0.92-0.95)	0.96 (0.95-0.98)
Past smoker	76,941 (27.9)	0.96 (0.95-0.97)	0.96 (0.95-0.98)
Non-smoker	126,497 (45.9)	1	1
Unknown	40,645 (14.8)	0.95 (0.94-0.97)	0.95 (0.93-0.96)
**Year group**			
2006-2007	58,606 (21.3)	1	1
2008-2009	60,212 (21.9)	0.99 (0.98-1.01)	0.99 (0.98-1.01)
2010-2011	59,183 (21.5)	1.00 (0.99-1.02)	1.00 (0.99-1.02)
2012-2013	55,099 (20.0)	0.99 (0.97-1.00)	0.99 (0.97-1.01)
2014-2015	42,477 (15.4)	0.95 (0.94-0.97)	0.95 (0.94-0.97)

Abbreviations: IRR – incidence rate ratio, CI – confidence interval.
Practice and woman are included as random effects terms in all models
Models exclude women with less than five weeks of follow-up information and missing Townsend score

For peer review only - http://bmjopen.bmj.com/site/about/guidelines.xhtml

Holly Smith, 06/01/20
Postnatal_check_manuscript

Table 3: Crude consultation rate per/ person-year, by characteristic

Characteristic	Number of consultations	Person-years	Rate of consultations per person-year (95% CI)
**Overall**	1,427,710	299,688	4.8 (4.8-4.8)
**Maternal age (years)**			
15-19	46,087	9,135	5.0 (5.0-5.1)
20-24	211,905	41,212	5.1 (5.1-5.2)
25-29	371,051	74,994	4.9 (4.9-5.0)
30-34	437,873	95,385	4.6 (4.6-4.6)
35-39	283,912	62,581	4.5 (4.5-4.6)
40-44	72,941	15,561	4.7 (4.7-4.7)
45-49	3,941	821	4.8 (4.7-5.0)
**Townsend Score quintile**			
1-least deprived	269,502	57,128	4.7 (4.7-4.7)
2	242,798	52,137	4.6 (4.6-4.7)
3	285,632	59,972	4.8 (4.7-4.8)
4	274,005	56,420	4.9 (4.8-4.9)
5-most deprived	212,965	42,609	5.0 (5.0-5.0)
Missing	142,808	31,422	4.5 (4.5-4.6)
**Mode of delivery**			
Vaginal delivery	511,769	73,251	7.0 (7.0-7.0)
Caesarean	176,199	22,743	7.7 (7.7-7.8)
Unknown	739,742	203,694	3.6 (3.6-3.6)
**Parity**			
First	655,616	144,425	4.5 (4.5-4.6)
Second	304,482	67,373	4.5 (4.5-4.5)
Third or higher	100,156	19,722	5.0 (5.0-5.1)
Unknown	367,456	68,168	5.4 (5.4-5.4)
**Smoking status**			
Current smoker	201,192	34,045	5.9 (5.9-5.9)
Past smoker	403,203	82,292	4.9 (4.9-4.9)
Non-smoker	656,620	139,161	4.7 (4.7-4.7)
Unknown	166,695	44,190	3.8 (3.8-3.8)
**Year group**			
2006-2007	302,645	61,803	4.9 (4.9-4.9)
2008-2009	318,827	64,356	5.0 (4.9-5.0)
2010-2011	315,752	64,396	4.9 (4.9-4.9)
2012-2013	285,875	60,974	4.7 (4.7-4.7)
2014-2015	204,611	48,159	4.2 (4.2-4.3)

Abbreviations: CI – confidence interval

For peer review only - http://bmjopen.bmj.com/site/about/guidelines.xhtml

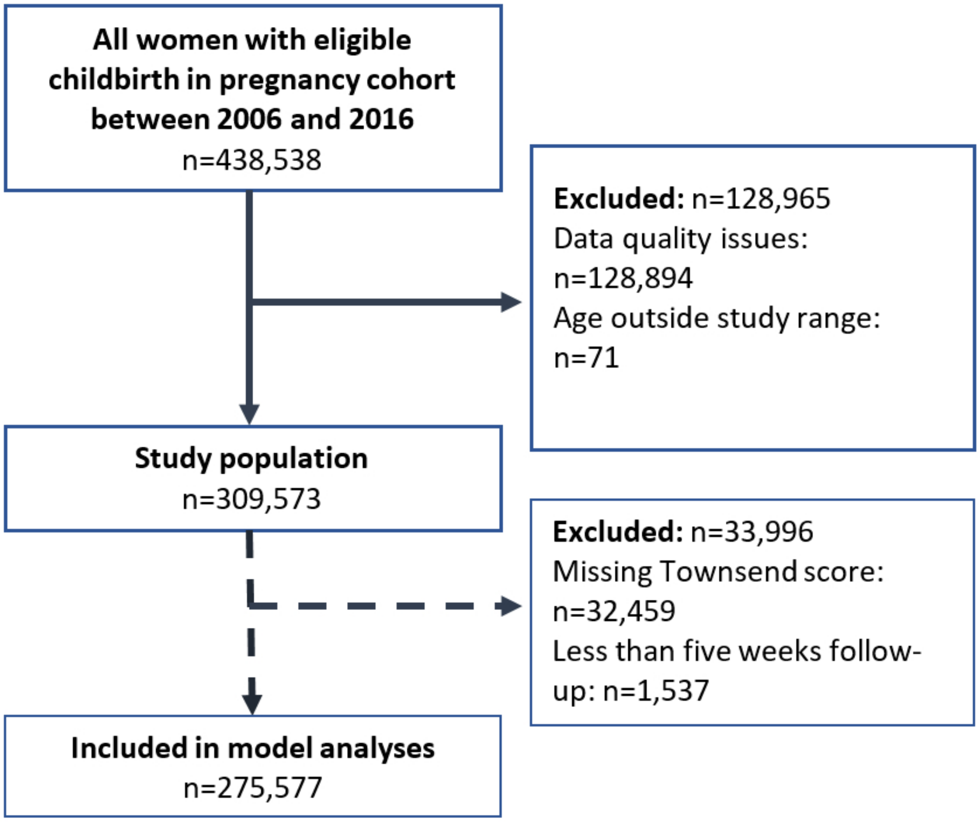

Figure 1: Flow diagram showing application of study inclusion and exclusion criteria

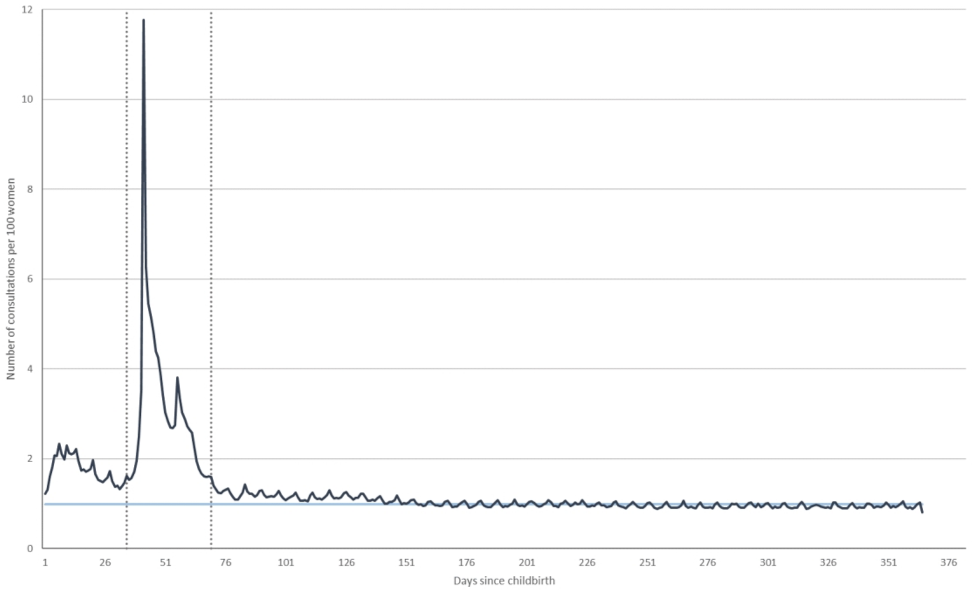

Figure 2: Women's consultation rate on each day in the first year following childbirth *Dotted lines indicate weeks 5 and 10, horizontal line indicates consultation rate after week 10.

Holly Smith, 06/01/20
Postnatal_check_supplementary

Supplementary materials

Likelihood of having a postnatal check using a more sensitive outcome definition (any consultation between weeks 5-10)

Characteristic	All women n	Consultation in weeks 5-10 n (%)	Consultation in weeks 5-10: Unadjusted: IRR (95% CI)	Consultation in weeks 5-10: Age & deprivation adjusted: IRR (95% CI)
**Overall**	309,573	243,516 (78.7)		
**Maternal age (years)**				
15-19	9,568	6,977 (72.9)	0.94 (0.91-0.96)	0.94 (0.92-0.97)
20-24	43,116	32,429 (75.2)	0.96 (0.95-0.98)	0.97 (0.95-0.98)
25-29	77,698	60,597 (78.0)	0.99 (0.98-1.00)	0.99 (0.98-1.00)
30-34	98,269	78,671 (80.1)	1	1
35-39	64,171	51,504 (80.3)	1.00 (0.98-1.01)	1.00 (0.98-1.01)
40-44	15,908	12,674 (79.7)	0.99 (0.97-1.01)	0.99 (0.97-1.01)
45-49	843	664 (78.8)	0.98 (0.90-1.06)	0.97 (0.90-1.06)
**Townsend Score quintile**				
1-least deprived	58,583	48,142 (82.2)	1	1
2	53,656	43,336 (80.8)	1.00 (0.98-1.01)	1.00 (0.98-1.01)
3	62,023	49,169 (79.3)	0.99 (0.97-1.00)	0.99 (0.98-1.00)
4	58,506	45,574 (77.9)	0.98 (0.96-0.99)	0.98 (0.97-1.00)
5-most deprived	44,346	32,729 (73.8)	0.95 (0.93-0.96)	0.95 (0.94-0.97)
Missing	32,459	24,566 (75.7)	Excluded	Excluded
**Mode of delivery**				
Vaginal delivery	75,506	63,533 (86.8)	1	1
Caesarean	23,426	20,074 (85.7)	1.03 (1.01-1.04)	1.02 (1.01-1.04)
Unknown	210,641	159,533 (75.7)	0.96 (0.94-0.98)	0.96 (0.94-0.97)
**Parity**				
First	149,639	118,998 (79.5)	1	1
Second	69,355	53,969 (77.8)	0.97 (0.96-0.98)	0.97 (0.96-0.98)
Third or higher	20,113	15,258 (75.9)	0.95 (0.93-0.96)	0.94 (0.92-0.95)
Unknown	70,466	55,291 (78.5)	0.96 (0.95-0.97)	0.95 (0.94-0.96)
**Smoking status**				
Current smoker	34,634	27,236 (78.6)	0.99 (0.98-1.01)	1.01 (0.99-1.02)
Past smoker**	85,592	66,542 (77.7)	0.97 (0.96-0.98)	0.97 (0.96-0.98)
Non-smoker	143,349	115,019 (80.2)	1	1
Unknown	45,998	34,719 (75.5)	0.93 (0.92-0.94)	0.93 (0.92-0.94)
**Year group**				
2006-2007	63,793	50,496 (79.2)	1	1
2008-2009	66,319	52,571 (79.3)	1.00 (0.98-1.01)	1.00 (0.98-1.01)
2010-2011	66,478	52,819 (79.5)	1.00 (0.99-1.02)	1.01 (0.99-1.02)
2012-2013	63,180	49,788 (78.8)	1.00 (0.99-1.02)	1.00 (0.99-1.02)
2014-2015	49,803	37,842 (76.0)	0.97 (0.96-0.99)	0.97 (0.96-0.99)

* Abbreviations: IRR – incidence rate ratio, CI – confidence interval.
**Practice and woman are included as random effects terms in all models
***Models exclude women with missing Townsend score

For peer review only - http://bmjopen.bmj.com/site/about/guidelines.xhtml

Holly Smith, 06/01/20
Postnatal_check_supplementary

For peer review only

For peer review only - http://bmjopen.bmj.com/site/about/guidelines.xhtml

STROBE Statement—Checklist of items that should be included in reports of ***cohort studies***

	Item No	Recommendation	Page No
**Title and abstract**	1	(*a*) Indicate the study's design with a commonly used term in the title or the abstract	1-2
		(*b*) Provide in the abstract an informative and balanced summary of what was done and what was found	2
**Introduction**			
Background/rationale	2	Explain the scientific background and rationale for the investigation being reported	4
Objectives	3	State specific objectives, including any prespecified hypotheses	4
**Methods**			
Study design	4	Present key elements of study design early in the paper	1-2
Setting	5	Describe the setting, locations, and relevant dates, including periods of recruitment, exposure, follow-up, and data collection	5-6
Participants	6	(*a*) Give the eligibility criteria, and the sources and methods of selection of participants. Describe methods of follow-up	5-6
		(*b*) For matched studies, give matching criteria and number of exposed and unexposed	
Variables	7	Clearly define all outcomes, exposures, predictors, potential confounders, and effect modifiers. Give diagnostic criteria, if applicable	6-7
Data sources/ measurement	8*	For each variable of interest, give sources of data and details of methods of assessment (measurement). Describe comparability of assessment methods if there is more than one group	5-7
Bias	9	Describe any efforts to address potential sources of bias	6
Study size	10	Explain how the study size was arrived at	5-8
Quantitative variables	11	Explain how quantitative variables were handled in the analyses. If applicable, describe which groupings were chosen and why	6-7
Statistical methods	12	(*a*) Describe all statistical methods, including those used to control for confounding	7
		(*b*) Describe any methods used to examine subgroups and interactions	7
		(*c*) Explain how missing data were addressed	6-7
		(*d*) If applicable, explain how loss to follow-up was addressed	5-7
		(*e*) Describe any sensitivity analyses	6-7
**Results**			
Participants	13*	(a) Report numbers of individuals at each stage of study—eg numbers potentially eligible, examined for eligibility, confirmed eligible, included in the study, completing follow-up, and analysed	8
		(b) Give reasons for non-participation at each stage	8
		(c) Consider use of a flow diagram	8, figure 1
Descriptive data	14*	(a) Give characteristics of study participants (eg demographic, clinical, social) and information on exposures and potential confounders	8
		(b) Indicate number of participants with missing data for each variable of interest	8, table1
		(c) Summarise follow-up time (eg, average and total amount)	8-9, table2
Outcome data	15*	Report numbers of outcome events or summary measures over time	8-9

For peer review only

Main results	16	(*a*) Give unadjusted estimates and, if applicable, confounder-adjusted estimates and their precision (eg, 95% confidence interval). Make clear which confounders were adjusted for and why they were included<br>(*b*) Report category boundaries when continuous variables were categorized<br>(*c*) If relevant, consider translating estimates of relative risk into absolute risk for a meaningful time period	8-9, all tables<br><br>6-7, tables<br>n/a
Other analyses	17	Report other analyses done—eg analyses of subgroups and interactions, and sensitivity analyses	8, supplementary material
**Discussion**			
Key results	18	Summarise key results with reference to study objectives	10
Limitations	19	Discuss limitations of the study, taking into account sources of potential bias or imprecision. Discuss both direction and magnitude of any potential bias	10
Interpretation	20	Give a cautious overall interpretation of results considering objectives, limitations, multiplicity of analyses, results from similar studies, and other relevant evidence	11-12
Generalisability	21	Discuss the generalisability (external validity) of the study results	11-12
**Other information**			
Funding	22	Give the source of funding and the role of the funders for the present study and, if applicable, for the original study on which the present article is based	13-14

*Give information separately for exposed and unexposed groups.

Note: An Explanation and Elaboration article discusses each checklist item and gives methodological background and published examples of transparent reporting. The STROBE checklist is best used in conjunction with this article (freely available on the Web sites of PLoS Medicine at http://www.plosmedicine.org/, Annals of Internal Medicine at http://www.annals.org/, and Epidemiology at http://www.epidem.com/). Information on the STROBE Initiative is available at http://www.strobe-statement.org.

For peer review only

BMJ Open

Postnatal checks and primary care consultations in the year following childbirth: an observational cohort study of 309,573 women in the UK, 2006-2016

Journal:	*BMJ Open*
Manuscript ID	bmjopen-2020-036835.R1
Article Type:	Original research
Date Submitted by the Author:	29-Jun-2020
Complete List of Authors:	Smith, Holly; University College London, Primary Care and Population Health<br>Saxena, Sonia; Imperial College London, School of Public Health;<br>Petersen, Irene; University College London Medical School, Department of Primary Care and Population health
**Primary Subject Heading**:	General practice / Family practice
Secondary Subject Heading:	Epidemiology, Obstetrics and gynaecology
Keywords:	Maternal medicine < OBSTETRICS, PRIMARY CARE, EPIDEMIOLOGY, REPRODUCTIVE MEDICINE

SCHOLARONE™
Manuscripts

I, the Submitting Author has the right to grant and does grant on behalf of all authors of the Work (as defined in the below author licence), an exclusive licence and/or a non-exclusive licence for contributions from authors who are: i) UK Crown employees; ii) where BMJ has agreed a CC-BY licence shall apply, and/or iii) in accordance with the terms applicable for US Federal Government officers or employees acting as part of their official duties; on a worldwide, perpetual, irrevocable, royalty-free basis to BMJ Publishing Group Ltd ("BMJ") its licensees and where the relevant Journal is co-owned by BMJ to the co-owners of the Journal, to publish the Work in this journal and any other BMJ products and to exploit all rights, as set out in our licence.

The Submitting Author accepts and understands that any supply made under these terms is made by BMJ to the Submitting Author unless you are acting as an employee on behalf of your employer or a postgraduate student of an affiliated institution which is paying any applicable article publishing charge ("APC") for Open Access articles. Where the Submitting Author wishes to make the Work available on an Open Access basis (and intends to pay the relevant APC), the terms of reuse of such Open Access shall be governed by a Creative Commons licence – details of these licences and which Creative Commons licence will apply to this Work are set out in our licence referred to above.

Other than as permitted in any relevant BMJ Author's Self Archiving Policies, I confirm this Work has not been accepted for publication elsewhere, is not being considered for publication elsewhere and does not duplicate material already published. I confirm all authors consent to publication of this Work and authorise the granting of this licence.

For peer review only

Holly Smith, 29/06/20
Postnatal_check_manuscript

Title:

Postnatal checks and primary care consultations in the year following childbirth: an observational cohort study of 309,573 women in the UK, 2006-2016

Authors:

Holly Christina Smith (0000-0001-6805-680X), Sonia Saxena (0000-0003-3787-2083), Irene Petersen (0000-0002-0037-7524).

Holly Christina Smith, PhD student, Department of Primary Care and Population Health, University College London, Royal Free Campus, Rowland Hill Street, London, NW3 2PF.

Sonia Saxena, Professor of Primary Care, School of Public Health, Faculty of Medicine, Imperial College London, 332 Reynolds Building, Charing Cross Campus, W6 8RP.

Irene Petersen, Professor of Epidemiology and Health Informatics, Department of Primary Care and Population Health, Institute of Epidemiology & Health, University College London, Royal Free Campus, Rowland Hill Street, London, NW3 2PF.

Correspondence to: Holly Christina Smith**:** Holly.dorning.18@ucl.ac.uk.

Word count:

3,614

For peer review only

For peer review only - http://bmjopen.bmj.com/site/about/guidelines.xhtml

Holly Smith, 29/06/20
Postnatal_check_manuscript

Abstract

Objective: To describe women's uptake of postnatal checks and primary care consultations in the year following childbirth.

Design: Observational cohort study using electronic health records.

Setting: UK primary care.

Participants: Women aged 16-49 years who had given birth to a single live infant recorded in The Health Improvement Network (THIN) primary care database in 2006-2016.

Main outcome measures: Postnatal checks and direct consultations in the year following childbirth.

Results: We examined 1,427,710 consultations in 309,573 women who gave birth to 241,662 children in 2006-2016. Of these women, 78.7% (243,516) had a consultation at the time of the postnatal check, but only 56.2% (174,061) had a structured postnatal check documented. Teenage women (aged 16 to 19 years) were 12% less likely to have a postnatal check compared with those aged 30-35 years (incidence rate ratio (IRR)=0.88, 95% CI: 0.85-0.91) and those living in the most deprived vs. least areas were 10% less likely (IRR=0.90, 95% CI: 0.88-0.92). Women consulted on average 4.8 times per women per year and 293,049 women (94.7%) had at least one direct consultation in the year after childbirth. Consultation rates were higher for those with a caesarean delivery (7.7 per women per year, 95% CI: 7.7-7.8). Consultation rates peaked during weeks 5-10 following birth (11.8 consultations/ 100 women) coinciding with the postnatal check.

Conclusions: Two in ten women did not have a consultation at the time of the postnatal and four in ten women have no record of receiving a structured postnatal check within the first ten weeks after giving birth. Teenagers and those from the most deprived areas are among the least likely to have a check. We estimate up to 350,400 women per year in the UK may be missing these opportunities for timely health promotion and to have important health needs identified following childbirth.

Keywords: Maternal Health, Postnatal Care, Primary Health Care, Electronic Health Records, Postpartum Period

For peer review only - http://bmjopen.bmj.com/site/about/guidelines.xhtml

Strengths and limitations of this study

- A major strength of this study is that it is among the largest population-based studies to date of postnatal care in the first 12 months.
- We drew on data from electronic health records which reflect real-world clinical practice in UK primary care.
- We are limited by what is recorded in a woman's electronic health record and genuine postnatal checks may have occurred in reality which were not accurately coded in primary care data.
- This study may underestimate the number of women who had a postnatal check compared with estimates based on survey data.

For peer review only - http://bmjopen.bmj.com/site/about/guidelines.xhtml

Introduction

Providing high quality comprehensive postnatal primary care is a global goal for improving maternal and child health in the first year of life [1]. In the United Kingdom (UK), nearly 800,000 women give birth each year [2] and every woman has access to midwives and health visitors for the first few days after delivery. They are then discharged to the care of their General Practitioner (GP) who invites them for a planned postnatal check 6-8 weeks after birth [3], as recommended by National Institute for Health and Care Excellence (NICE)[4] and the World Health Organisation (WHO) as part of routine postnatal care[5]. The maternal postnatal check provides a unique and timely opportunity for new mothers and health care professionals to evaluate their physical and mental health and assess how women are recovering after pregnancy and birth[6]. The postnatal check is also a point where women and primary care health professionals can discuss breastfeeding, postpartum contraception, smoking cessation, return to physical activity, and dietary advice, particularly after gestational weight gain. GPs also play a role in supporting parents to cope with managing day to day care and minor illness of infants and identify safeguarding concerns for new mothers and their babies.

The postnatal period is typically defined as the first six weeks after childbirth[7] and previous studies estimate 47% to 83% of women will report at least one health problem around eight weeks postpartum [8-10]. Historically, it was anticipated women would recover from pregnancy within this time [11]; however, there is increasing evidence that women have ongoing health needs throughout the first year [12] and even longer [13 14]. For example, up to 5% of women will require ongoing management of medical complications of gestational diabetes[15]; and others may need support with postpartum conditions such as postnatal depression which occurs in 1 in 6 [16]. Previous research may underestimate maternal morbidity as many women do not report symptoms or may be reluctant to seek support after childbirth [17].

From previous cross-sectional surveys from 1995 and 2010-2014, it would appear that up to 90% of women attend their postnatal checks, but selection bias of survey participants may overestimate this figure [18-20]. We found no contemporary studies showing patterns of primary care use for women following childbirth. The aim of this observational cohort study was to examine the prevalence of postnatal checks, explore factors associated with having a postnatal check and primary care consultations for women in the first year after childbirth.

For peer review only - http://bmjopen.bmj.com/site/about/guidelines.xhtml

Methods

UK healthcare

In the UK, healthcare is free at the point of delivery for all residents as part of the National Health Service (NHS). Primary care is typically the first point of contact and is largely delivered by GPs and other health care professionals (nurses and health visitors) within a practice. Information about patients and their health are collected during primary care consultations. This information is primarily used for clinical care but is also widely used for research through large healthcare databases.

Data source

We used one of the largest UK primary care databases, The Health Improvement Network (THIN) database. As of December 2016, THIN contained anonymised electronic health records for 16 million registered patients from 730 practices across the UK [21]. The database contains patient-level information on: demographics, prescribing, symptoms, procedures, prevention, lifestyle factors and diagnostics. Consultations can be linked to give a comprehensive picture of someone's care. Diagnostic and symptomatic information are categorised using Read codes, a hierarchical coding system [22]. Additional Health Data codes classify prevention and lifestyle information. Socioeconomic information is captured through Townsend score which provides a measure of material deprivation based on: where a person lives, unemployment, car ownership, home ownership and household overcrowding [23]. THIN is broadly representative of the UK population in terms of demographics, chronic disease and mortality. However, there is an over-representation of more affluent people as there is a greater proportion of people who live in less deprived areas contributing to THIN database compared to the UK general population [24].

Study population

A cohort of women who have given birth to a single live infant has previously been identified within THIN [25]. Births and date of childbirth were identified using a combination of an antenatal record, delivery record, postnatal care record, date of last menstrual period, or birth of a child matched to mother's record. This pre-existing cohort of women excluded more complex births, including multiple deliveries (twins, triplets etc.) and those with a known miscarriage, termination or stillbirth, so it is not possible to include these women in our study. This cohort contains information on approximately 650,000 pregnancies/childbirths in 1990-2016. In this study, we included women aged 15 to 49 years who gave birth between 1st January 2006 and 31st December 2015 from this

For peer review only - http://bmjopen.bmj.com/site/about/guidelines.xhtml

cohort. Data quality criteria were also applied whereby practices which did not have acceptable computer use (ACU) or acceptable mortality rates (AMR) by the date of childbirth were excluded. ACU is the date a practice was continuously entering on average at least two therapy records, one medical record and one additional health data record per patient per year [26]; and AMR is the date a practice has comparable mortality rates to the rest of the UK, given the size and demographics of the practice [27]. Women who had been registered at a practice for less than six months were also excluded. It was possible for women to have multiple childbirths in this study. Women were followed-up for 12 months to identify their primary use after each childbirth, censoring for maternal death or practice transfer.

Definition of variables

Postnatal check

We identified a postnatal check as any consultation at the time of the check (typically weeks 6-8) which had a specific Read code (beginning with '62R' or '62S') or Additional Health Data code ('1044100000' or '1044000000') identifying it as postnatal visit and/or check. Some women may receive this check slightly earlier or later, and on reviewing the data this window was expanded to weeks 5-10 after birth to include a peak in consultations. We identified substantial variation in the use of these codes by practices and change over time (data not shown). Therefore, we used a second and more sensitive approach where we assumed any consultation at the time of the routine appointment (weeks 5-10) was an opportunity for a postnatal check. The results of this second approach are included in supplementary materials.

Consultations

A primary care consultation was defined as any direct contact between a patient and a healthcare professional taking place: in practice, in a patient's home or by telephone. It was assumed only one consultation took place each day for each woman, therefore multiple records on the same date were grouped.

Patient and childbirth characteristics

We stratified our analysis by maternal age, number of births, Townsend score (described previously), smoking status, year (two-year bands) and mode of delivery. Women were assigned to a five-year bands according to their age. We used information in the mother's records as well as children registered within the same household at the time of birth to assign number of births (categorised as: First, Second, Third or higher, or Unknown). We used Townsend score quintiles whereby each woman is assigned to one of five groups of deprivation, from least to most deprived. We assigned

For peer review only

women's smoking status as 'current smoker' (record of smoking at any time in the year after childbirth), 'past smoker' (record of smoking or being an ex-smoker in the two years prior to childbirth and not a current smoker), 'non-smoker' or 'unknown'. Mode of delivery was determined using the identifying pregnancy/childbirth Read codes and was broadly grouped into 'caesarean', 'vaginal' and 'unknown' based on classifications developed previously [25].

Statistical analysis

A table was derived to show characteristics of women at each childbirth. The crude consultation rate was calculated as the total number of consultations per total person-years, stratified by characteristics. To explore variation across the first year, consultation rate was calculated as the number of consultations on each day with number of women registered with a practice on that day as the denominator. Women who died or transferred practice were censored from the denominator each day. To examine those who had a postnatal check, firstly we calculated the crude proportion of women with the outcome in each patient strata. To explore variation by characteristic in more detail, we examined the likelihood of having a postnatal check between 5-10 weeks in women with at least five weeks follow-up and complete deprivation (Townsend score) information. We developed mixed-effects Poisson models to estimate how the likelihood of having a postnatal check between weeks 5-10 varied by maternal age, Townsend score, mode of delivery, number of births, smoking status and year. Three models were developed: unadjusted, age-adjusted and age-deprivation adjusted. Practice and woman (as women can have multiple childbirths) were included as random effects terms, and the log of follow-up time (between weeks 5-10) was included as an offset. All analyses were conducted using Stata V.16 (StataCorp, College Station, Texas, USA).

Patient and public involvement

Two public panels interested in primary care research provided feedback on the study outline. The groups were supportive of the idea to identify attendance of postnatal checks, they suggested we clearly identify groups who do not attend and to examine differences in a woman's first vs subsequent childbirths. As a result, we have included additional analysis exploring attendance by patient characteristics and number of births.

For peer review only - http://bmjopen.bmj.com/site/about/guidelines.xhtml

Results

Participants

Between 1 January 2006 and 31 December 2016, 438,538 pregnancies/childbirths were identified in the pregnancy cohort within THIN data. Study inclusion and exclusion criteria were applied to these records which resulted in a final sample of 309,573 childbirths (Figure 1).

Characteristics of women

We identified 309,573 childbirths in this study related to 241,662 women. At childbirth, a third of the women were aged 30-34 years (31.7%). There were 21.1% in the least deprived Townsend quintile compared 16.0% in the most deprived (which is similar to the overall distribution in THIN [24]). Three quarters of women had a vaginal delivery and the rest had a caesarean birth (76.3% vs 23.7%). Of these, nearly half were a first birth (48%) and 22% were a second birth. Half of women were non-smokers (46.3%), compared to 11.2% being current smoker (Table 1).

Postnatal check

Overall, just over half of the women in our study (56%) had a postnatal check, i.e. 44% had no such records (Table 1). In this crude analysis, younger women and those from the most deprived areas were less likely to have a postnatal check (48% of those aged 15-19 years vs 59.5% of those aged 35-39 years; and 47.7% of those from the most deprived area vs 62.7% from the least).

After excluding those with less than five weeks of follow-up information, missing deprivation information, 275,577 women were included in additional analysis (Figure 1). Those aged 15-19 years were 12% less likely (incidence rate ratio (IRR)=0.88, 95% CI: 0.85-0.91) to have a postnatal check between weeks 5-10 relative to women aged 30-35 years (Table 2). Similarly, women from the most deprived areas were 10% less likely (IRR=0.90, 95% CI: 0.88-0.92) to have a postnatal check relative to those from the least deprived areas. Differences across other characteristics were less pronounced; and, adjusting for age, and age and deprivation, had little impact on differences across these other characteristics. The same trend across age and deprivation was identified when using a more sensitive approach to identify a potential postnatal check (any consultation between weeks 5-10), but with a higher proportion of women (78.7%) having a consultation (see supplementary material).

A small proportion of women in our study 18,723 (6.0%) had a consultation in the first four weeks but did not have a consultation in weeks 5-10 (see supplementary material). This compares to a

For peer review only - http://bmjopen.bmj.com/site/about/guidelines.xhtml

For peer review only

much greater proportion of women 89,605 (28.9%) who had both an early consultation and one in weeks 5-10.

Primary care consultations

Following the 309,573 childbirths, the majority (94.7%, n=293,049) of women had at least one direct consultation in the year after childbirth. A total of 1,427,710 direct consultations were identified, with women consulting on average 4.8 times/person-year (Table 3). The largest differences in consultation rate compared to the average is seen in those who had a caesarean delivery (7.7/ person-year, 95% CI: 7.7-7.8) and in current smokers (5.9/ person-year, 95% CI: 5.9-5.9). Consultation rate decreased over time, from 4.9/ person-year (95% CI: 4.9-4.9) in 2006-2007 to 4.2/ person-year in 2014-2015 (4.2-4.3). Consultation rates were broadly similar across other characteristics.

Across the first year the consultation rate was highest between weeks 5-10, with a peak of 11.8 consultations/ 100 women in week 6, coinciding with the postnatal check. Following this, the consultation rate fell to an average of 1 consultation/ 100 women (Figure 2).

For peer review only

For peer review only - http://bmjopen.bmj.com/site/about/guidelines.xhtml

Discussion

Main findings

We found that eight in 10 women had a consultation at the time of the postnatal check; however, only half of women had a record of receiving a structured postnatal check which means four in ten women (44%) may have missed out. Teenage women (aged 15-19 years) were 12% less likely to have a postnatal check compared with older women aged 30-35 years, and those living in the most deprived areas were 10% less likely compared to women from least deprived areas. Women consulted on average 4.8 times per year in the year after childbirth and 94.7% of women had at least one consultation in the year after childbirth. Those who had a caesarean delivery and smokers had higher than average consultation rates (7.7 times per women per year and 5.9 times per women per year respectively). Consultation rates decreased over time (from 4.9 time per woman per year in 2006-2007 to 4.2 times in 2014-2015). Across the first year, the consultation rate is highest in week six with a peak of 11.8 consultations/ 100 women, which coincides with the postnatal check, after week ten the consultation rate is flat with 1 consultation/ 100 women on each day.

Study strengths and weaknesses

This is among the largest (299,688 person years) population-based studies to date of postnatal care in the first 12 months. The use of electronic health records provides a reflection of real-world clinical practice allowing us to explore use of the postnatal check in a broad population (not reliant on patient participation). As with all studies of electronic health records however, we are limited by what has been recorded in a woman's record which could mean patient, birth and consultation characteristics may be missing or not accurate. We also recognise the limitations in using Read codes/AHD codes only to identify a postnatal check as there is variation in the use of these by general practice and genuine checks may not be coded as such in primary care data. To account for this, we repeated our analysis using a more sensitive definition of a postnatal check (any consultation in week 5-10). While we found a larger proportion (78.7%) having been in contact with primary care, the overall trends in terms of the sociodemographic information was consistent between the two approaches.

Findings in relation to previous studies

The characteristics of women in our cohort are broadly similar to all women who give birth in terms of age and mode of delivery in a comparison year of 2017[28,29]. However, our cohort has a lower proportion of women from more deprived areas compared to all births in England and Wales, which

For peer review only - http://bmjopen.bmj.com/site/about/guidelines.xhtml

limits the generalisability of our findings. We identified that those from more deprived areas were less likely to have a postnatal check which supports previous findings[20]. This may mean we overestimate the proportion of women who have a postnatal check compared to all women who give birth in the UK. Previous studies estimate that 85% to 91% of women in England have a postnatal check [18 19]; however only 56% of women in our study had evidence of having one. We would expect our estimates to be lower as we used electronic health records which capture a broader picture of real-world practice compared with previous studies that may have been subject to selection and recall bias. When using a more sensitive approach, where we considered any consultation in weeks 5-10 as evidence of a postnatal check, our findings are closer to those of previous studies, although we are aware that not all of these consultations would have covered topics meant for the postnatal check. It is likely the true number having a check lies between our two estimates. The consultation rate of 4.8 per person per year identified in our study is comparable to that found by others, when taking age, sex and reason for consultation into account[30].

There are several possible explanations for our findings of a low uptake of postnatal checks. It is possible that women do not want or feel they need advice from GPs; or invitations from the GP are not taken up either because women do not respond to them, or may find it difficult to access appointments. Alternatively a lack of recording in electronic health records may explain the apparently low rate of postnatal checks.

Implications of findings and future research recommendations

It is encouraging to find that the majority of women return to primary care at least once in the year after childbirth; however, it is concerning that four in ten women did not have a structured postnatal check documented and that consultation rates have declined over time. In UK, approximately 800,000 women give birth each year[2], our estimates (44%) then suggest that up to 350,400 women may be missing this key check. The postnatal period is a potentially vulnerable time for women and there could be serious consequences to not identifying women at risk of poor health or harm after childbirth[31]. The postnatal check has been shown to be a key contact to identify serious health needs such as postnatal depression, which affects 1 in 6 women after childbirth. [25] It also provides protected time and opportunities to improve women's health and wellbeing through preventative intervention such as timely access to contraception, advice about weight management or diet following gestational weight gain, or support to stop smoking can be given [4]. Our finding that younger women and those from more deprived areas are less likely to have a check is particularly important as they may be most likely to benefit. For example, contraceptive uptake is particularly

For peer review only - http://bmjopen.bmj.com/site/about/guidelines.xhtml

low in younger and more deprived groups[32], and offering timely access through the check could lead to fewer unwanted or repeat pregnancies for these women.

Our findings suggest practices may need to implement systems for follow up of women who have declined or missed a postnatal check. There is a need for better promotion of the benefits of attending the postnatal check at other times in the maternity pathway; such as during midwife or health visitor appointments, in hospital or birth units, or at other GP maternity and baby check-ups. Additionally, there are currently no known financial or quality-based incentives to document primary care activity in the postnatal period. This could lead to variation in services and underreporting of activity. It is vital to improve the documentation of this care to more accurately understand women's use of the postnatal check, more broadly their health needs and service use after childbirth, and ultimately improve care. This is particularly important as we identified that postnatal consultation rates declined over time. We recommend more research to explore the reasons behind the low uptake of postnatal checks and variation in consultation rates.

In this study our focus was on who had a postnatal check, and while NICE outlines the content of these appointments, few studies have explored what health needs are covered in actuality. This should be explored through further research to better understand what content and delivery are most effective for women and if attending a postnatal check leads to better outcomes. Current NICE guidance recommends the postnatal check take place 6 weeks after childbirth within primary care. There has been some evaluation of this timing and frequency,[33,34] and in particular how this relates to the early postnatal care women receive from midwives. Further high-quality studies are needed to determine the most effective timing of postnatal care consultations. It is also important to examine the accuracy of postnatal care in electronic health records and explore ways to improve this in future studies. Lastly, our study focused on the postnatal care use of women who had given birth to a single live infant only. Complex births, such as multiple deliveries or stillbirths are relatively rare (15.9 out of every 1,000 women giving birth in England and Wales had a multiple birth in 2016 and 4.4 per 1,000 births were a stillbirth)[35]. It is expected that these women would receive additional follow-up in specialist care and so would not represent the usual pathway back to primary care services. However, this has not been well investigated and future studies should explore if these women have different experiences of postnatal care. Furthermore, future studies could explore the differences in postnatal care use by ethnicity, country of birth, language spoken and refugee or asylum seeker status.

Conclusion

For peer review only - http://bmjopen.bmj.com/site/about/guidelines.xhtml

Two in ten women had no consultation at the time of the postnatal check and four in ten women have no record of receiving a structured postnatal check within the first ten weeks after giving birth, this is despite the majority of women returning to primary care at least once in the year after childbirth. Teenagers and those from the most deprived areas are among the least likely to have a check. We estimate up to 350,400 women per year in the UK may be missing these opportunities for timely health promotion and to have important health needs identified after childbirth.

For peer review only

For peer review only - http://bmjopen.bmj.com/site/about/guidelines.xhtml

Acknowledgements

We are very grateful to members of the public who generously gave their time to feedback on the outline of this study, in particular we are grateful to the many women who have shared their experiences of childbirth and beyond, and who serve as a continued source of inspiration for this research. We are also appreciative for the support of our funders.

Footnotes

Authors contributions

This study was designed and conceived by HCS, SS and IP. HCS conducted the data analysis and wrote the first draft of this manuscript. SS and IP made comments on the manuscript. HCS and IP had full access to the database and guarantee this manuscript is an honest, accurate, and transparent account of the study being reported; that no important aspects of the study have been omitted; and that any discrepancies from the study as planned have been explained.

Funding

This study is funded by the National Institute for Health Research (NIHR) School for Primary Care Research (SPCR) (project reference 549832). The views expressed are those of the authors and not necessarily those of the NIHR or the Department of Health and Social Care.

SS is funded by the National Institute for Health Research (NIHR) School for Public Health Research (SPHR) and NIHR Northwest London Applied Research Collaboration (ARC). The School for Public Health Imperial College London is also grateful for support from the Imperial NIHR Biomedical Research Centre.

The funders had no role in study design, data collection and analysis, decision to publish, or preparation of the manuscript.

Data sharing statement

No additional data are available.

Competing interests

All authors have completed the ICMJE uniform disclosure form at www.icmje.org/coi_disclosure.pdf and declare: HCS reports grants from National Institute of Health Research School for Primary Care Research and SS reports grants from NIHR School for Public Health Research, grants from NIHR NW

For peer review only - http://bmjopen.bmj.com/site/about/guidelines.xhtml

London Applied Research Collaboration, during the conduct of the study; grants from The Daily Mile Foundation Charity, outside the submitted work; no other relationships or activities that could appear to have influenced the submitted work.

Ethical approval and data access

Approval was received from the Scientific Review Committee on 10/04/2019 (THIN protocol number: 19THIN013). THIN is a registered trademark of Cegedim SA in the UK and other countries. Reference made to THIN database is intended to be descriptive of the data asset licensed by IQVIA. This work uses de-identified data provided by patients as a part of their routine primary care.

Copyright/license for publication

The Corresponding Author HCS has the right to grant on behalf of all authors and does grant on behalf of all authors, a worldwide licence to the Publishers and its licensees in perpetuity, in all forms, formats and media (whether known now or created in the future), to i) publish, reproduce, distribute, display and store the Contribution, ii) translate the Contribution into other languages, create adaptations, reprints, include within collections and create summaries, extracts and/or, abstracts of the Contribution, iii) create any other derivative work(s) based on the Contribution, iv) to exploit all subsidiary rights in the Contribution, v) the inclusion of electronic links from the Contribution to third party material where-ever it may be located; and, vi) licence any third party to do any or all of the above.

As our data are anonymised it is not possible to disseminate our findings to study participants.

For peer review only

References

1. United Nations. Transforming our world: The 2030 agenda for sustainable development, 2015.
2. Office for National Statistics. Births by mothers' usual area of residence in the UK [Data file]. Office for National Statistics, London, 2016. [cited 12 Dec 2019]. Avaliable from: https://www.ons.gov.uk/peoplepopulationandcommunity/birthsdeathsandmarriages/livebirths/datasets/birthsbyareaofusualresidenceofmotheruk.
3. NHS Choices. Your 6-week postnatal check [Available from: https://www.nhs.uk/conditions/pregnancy-and-baby/postnatal-check/ accessed 20/12/2018.
4. National Institute for Health and Care Excellence. Postnatal care up to 8 weeks after birth. NICE Clinical Guideline 37, 2006. https://www.nice.org.uk/guidance/cg37.
5. World Health Organisation, United Nations Population Fund, Unicef. Pregnancy, Childbirth, Postpartum and Newborn Care: A guide for essential practice. Third ed. Geneva, 2015.
6. Jakes AD, Oakeshott P, Bick D. The maternal six week postnatal check. *BMJ* 2019;367:l6482. doi: 10.1136/bmj.l6482 [published Online First: 2019/12/04]
7. World Health Organisation. WHO Technical Consultation on Postpartum and Postnatal Care, 2010.
8. Glazener CM, Abdalla M, Stroud P, et al. Postnatal maternal morbidity: extent, causes, prevention and treatment. *Br J Obstet Gynaecol* 1995;102(4):282.
9. Thompson JF, Roberts CL, Currie M, et al. Prevalence and persistance of health problems after childbirth: associations with parity and method of birth. *Birth* 2002;29(2):83-94.
10. MacArthur C, Lewis M, Knox EG. Health after childbirth. *Br J Obstet Gynaecol* 1991;98:1193-204.
11. Albers LL. Health problems after childbirth. *Journal of Midwifery & Women's Health* 2000;45(1):55-57.
12. Ansara D, Cohen MM, Gallop R, et al. Predictors of women's physical health problems after childbirth. *Journal of Psychosomatic Obstetrics & Gynecology* 2009;26(2):115-25. doi: 10.1080/01443610400023064
13. Dahlen HG. Urinary incontinence: where to now? *BJOG* 2016;123(6):1030. doi: 10.1111/1471-0528.13427 [published Online First: 2015/05/27]
14. MacArthur C, Wilson D, Herbison P, et al. Faecal incontinence persisting after childbirth: a 12 year longitudinal study. *BJOG* 2013;120(2):169-79. doi: 10.1111/1471-0528.12039 [published Online First: 2012/11/30]
15. National Institute for Health and Care Excellence. Diabetes in pregnancy: management from preconception to the postnatal period. NICE Guideline No. 3, 2015. https://www.nice.org.uk/guidance/ng3.
16. Shorey S, Chee CYI, Ng ED, et al. Prevalence and incidence of postpartum depression among healthy mothers: A systematic review and meta-analysis. *J Psychiatr Res* 2018;104:235-48. doi: 10.1016/j.jpsychires.2018.08.001 [published Online First: 2018/08/17]
17. Chou D, Tuncalp O, Firoz T, et al. Constructing maternal morbidity - towards a standard tool to measure and monitor maternal health beyond mortality. *BMC Pregnancy Childbirth* 2016;16:45. doi: 10.1186/s12884-015-0789-4 [published Online First: 2016/03/05]
18. Bick DE, MacArthur C. Attendance, content and relevance of the six week postnatal examination. *Midwifery* 1995(11):69-73.
19. Redshaw M, Heikkila K. Delivered with care: a national survey of women's experience of maternity care 2010: National Perinatal Epidemiology Unit, 2010.
20. Redshaw M, Henderson J. Safely delivered: a national survey of women's experiences of maternity care 2014: National Perinatal Epidemiology Unit, 2015.
21. Quintiles IMS. The Health Improvement Network Data Guide for Researchers, 2017.
22. Booth N. What are the Read Codes? *Health Libr Rev* 1994;11:177-82.

For peer review only - http://bmjopen.bmj.com/site/about/guidelines.xhtml

23. Townsend P, Phillimore P, Beattie A. Health and deprivation: inequality and the north. London: Routledge 1989.

24. Blak BT, Thompson M, Dattani H, et al. Generalisability of The Health Improvement Network (THIN) database: demographics, chronic disease prevalence and mortality rates. *Inform Prim Care* 2011;19:251-5.

25. Petersen I, McCrea RL, Sammon CJ, et al. Risks and benefits of psychotropic medication in pregnancy: cohort studies based on UK electronic primary care health records. *Health Technol Assess* 2016;20(23):1-176. doi: 10.3310/hta20230 [published Online First: 2016/04/01]

26. Horsfall L, Walters K, Petersen I. Identifying periods of acceptable computer usage in primary care research databases. *Pharmacoepidemiol Drug Saf* 2013;22(1):64-9. doi: 10.1002/pds.3368 [published Online First: 2012/11/06]

27. Maguire A, Blak BT, Thompson M. The importance of defining periods of complete mortality reporting for research using automated data from primary care. *Pharmacoepidemiol Drug Saf* 2009;18(1):76-83. doi: 10.1002/pds.1688 [published Online First: 2008/12/10]

28. Office for National Statistics. Births by Parents' Characteristics, England and Wales [data file]. https://www.ons.gov.uk/peoplepopulationandcommunity/birthsdeathsandmarriages/livebirths/datasets/birthsbyparentscharacteristics. Published 2017. Accessed June 18, 2020.

29. NHS Digital. NHS Maternity Statistics, 2017-18: Hospital Episode Statistics [data file]. http://digital.nhs.uk/pubs/maternity1718. Published 2018. Accessed June 18, 2020.

30. Wang Y, Hunt K, Nazareth I, et al. Do men consult less than women? An analysis of routinely collected UK general practice data. *BMJ Open* 2013;3(8) doi: 10.1136/bmjopen-2013-003320

31. Knight M, Bunch K, Tuffnell D, et al. Saving Lives, Improving Mothers' Care: Lessons learned to inform maternity care from the UK and Ireland Confidential Enquiries into Maternal Deaths and Morbidity 2014–16, 2018.

32. National Institute for Health and Care Excellence. Contraceptive services for under 25s. NICE Public health guideline No. 51, 2014. www.nice.org.uk/guidance/ph51.

33. Gunn J, Lumley J, Chondros P, Young D. Does an early postnatal check-up improve maternal health: Results from a randomised trial in Australian general practice. *BJOG An Int J Obstet Gynaecol*. 1998;105(9):991-997. doi:10.1111/j.1471-0528.1998.tb10263.x

34. MacArthur C, Winter HR, Bick DE, et al. Effects of redesigned community postnatal care on womens' health 4 months after birth: a cluster randomised controlled trial. *Lancet*. 2002;359(9304):378-385. doi:10.1016/S0140-6736(02)07596-7

35. Office for National Statistics. Birth Characteristics in England and Wales [data file]. https://www.ons.gov.uk/peoplepopulationandcommunity/birthsdeathsandmarriages/livebirths/datasets/birthcharacteristicsinenglandandwales. Published 2016. Accessed June 18, 2020.

For peer review only - http://bmjopen.bmj.com/site/about/guidelines.xhtml

Holly Smith, 29/06/20
Postnatal_check_manuscript

Tables

Table 1: Characteristics of women who have given birth to a single live infant and proportion of those women with a structured postnatal check 5-10 weeks after childbirth

Characteristic	All women n	Record of postnatal check in weeks 5-10 n (% across the row)
**Overall**	309,573	174,061 (56.2)
**Maternal age (years)**		
15-19	9,568	4,599 (48.1)
20-24	43,116	21,763 (50.5)
25-29	77,698	42,417 (54.6)
30-34	98,269	57,308 (58.3)
35-39	64,171	38,154 (59.5)
40-44	15,908	9,347 (58.8)
45-49	843	473 (56.1)
**Townsend Score quintile**		
1-least deprived	58,583	36,752 (62.7)
2	53,656	32,326 (60.3)
3	62,023	35,413 (57.1)
4	58,506	31,601 (54.0)
5-most deprived	44,346	21,138 (47.7)
Missing	32,459	16,831 (51.9)
**Mode of delivery**		
Vaginal delivery	75,506	46,634 (61.8)
Caesarean	23,426	14,384 (61.4)
Unknown	210,641	113,043 (53.7)
**Number of births**		
First	149,639	84,010 (56.1)
Second	69,355	39,269 (56.6)
Third or higher	20,113	10,781 (53.6)
Unknown	70,466	40,001 (56.8)
**Smoking status**		
Current smoker	34,634	18,199 (52.6)
Past smoker	85,592	47,464 (55.5)
Non-smoker	143,349	82,420 (57.5)
Unknown	45,998	25,978 (56.5)
**Year group**		
2006-2007	63,793	36,863 (57.8)
2008-2009	66,319	38,124 (57.5)
2010-2011	66,478	37,897 (57.0)
2012-2013	63,180	34,896 (55.2)
2014-2015	49,803	26,281 (52.8)

For peer review only - http://bmjopen.bmj.com/site/about/guidelines.xhtml

Table 2: Mixed-effects Poisson estimates of the likelihood of having a postnatal check for women who had given birth to a single live infant by age, Townsend score, mode of delivery, number of births, smoking status and year group; unadjusted, and adjusted for age and deprivation

Characteristic	n (%)	Record of postnatal check in weeks 5-10	
		Unadjusted: IRR (95% CI)	Age & deprivation adjusted: IRR (95% CI)
**Overall**	275,577		
**Maternal age (years)**			
15-19	8,704 (3.2)	0.88 (0.85-0.91)	0.89 (0.87-0.92)
20-24	38,503 (14.0)	0.92 (0.91-0.94)	0.93 (0.92-0.95)
25-29	68,751 (25.0)	0.97 (0.96-0.98)	0.97 (0.96-0.99)
30-34	86,889 (31.5)	1	1
35-39	57,533 (20.9)	1.00 (0.99-1.02)	1.00 (0.99-1.01)
40-44	14,428 (5.2)	0.99 (0.97-1.01)	0.99 (0.97-1.01)
45-49	769 (0.3)	0.96 (0.87-1.05)	0.96 (0.87-1.05)
**Townsend Score quintile**			
1-least deprived	58,304 (21.2)	1	1
2	53,370 (19.4)	0.99 (0.98-1.01)	1.00 (0.98-1.01)
3	61,681 (22.4)	0.97 (0.95-0.98)	0.97 (0.96-0.99)
4	58,165 (21.1)	0.95 (0.93-0.97)	0.96 (0.95-0.98)
5-most deprived	44,057 (16.0)	0.90 (0.88-0.92)	0.92 (0.90-0.93)
**Mode of delivery**			
Vaginal delivery	68,202 (76.6)	1	1
Caesarean	20,828 (23.4)	1.02 (1.00-1.04)	1.01 (0.99-1.03)
Unknown	186,547	0.99 (0.97-1.01)	0.99 (0.97-1.01)
**Number of births**			
First	132,164 (48.0)	1	1
Second	62,535 (22.7)	0.99 (0.98-1.01)	0.98 (0.97-0.99)
Third or higher	18,504 (6.7)	0.95 (0.93-0.97)	0.94 (0.92-0.96)
Unknown	62,374 (22.6)	0.96 (0.95-0.97)	0.95 (0.94-0.96)
**Smoking status**			
Current smoker	31,494 (11.4)	0.94 (0.92-0.95)	0.96 (0.95-0.98)
Past smoker	76,941 (27.9)	0.96 (0.95-0.97)	0.96 (0.95-0.98)
Non-smoker	126,497 (45.9)	1	1
Unknown	40,645 (14.8)	0.95 (0.94-0.97)	0.95 (0.93-0.96)
**Year group**			
2006-2007	58,606 (21.3)	1	1
2008-2009	60,212 (21.9)	0.99 (0.98-1.01)	0.99 (0.98-1.01)
2010-2011	59,183 (21.5)	1.00 (0.99-1.02)	1.00 (0.99-1.02)
2012-2013	55,099 (20.0)	0.99 (0.97-1.00)	0.99 (0.97-1.01)
2014-2015	42,477 (15.4)	0.95 (0.94-0.97)	0.95 (0.94-0.97)

Abbreviations: IRR – incidence rate ratio, CI – confidence interval.
Practice and woman are included as random effects terms in all models
Models exclude women with less than five weeks of follow-up information and missing Townsend score

For peer review only - http://bmjopen.bmj.com/site/about/guidelines.xhtml

Table 3: Crude consultation rate in the first year after childbirth of women who had given birth to a single live infant per/ person-year, by characteristic

Characteristic	Number of consultations	Person-years	Rate of consultations per person-year (95% CI)
**Overall**	1,427,710	299,688	4.8 (4.8-4.8)
**Maternal age (years)**			
15-19	46,087	9,135	5.0 (5.0-5.1)
20-24	211,905	41,212	5.1 (5.1-5.2)
25-29	371,051	74,994	4.9 (4.9-5.0)
30-34	437,873	95,385	4.6 (4.6-4.6)
35-39	283,912	62,581	4.5 (4.5-4.6)
40-44	72,941	15,561	4.7 (4.7-4.7)
45-49	3,941	821	4.8 (4.7-5.0)
**Townsend Score quintile**			
1-least deprived	269,502	57,128	4.7 (4.7-4.7)
2	242,798	52,137	4.6 (4.6-4.7)
3	285,632	59,972	4.8 (4.7-4.8)
4	274,005	56,420	4.9 (4.8-4.9)
5-most deprived	212,965	42,609	5.0 (5.0-5.0)
Missing	142,808	31,422	4.5 (4.5-4.6)
**Mode of delivery**			
Vaginal delivery	511,769	73,251	7.0 (7.0-7.0)
Caesarean	176,199	22,743	7.7 (7.7-7.8)
Unknown	739,742	203,694	3.6 (3.6-3.6)
**Number of births**			
First	655,616	144,425	4.5 (4.5-4.6)
Second	304,482	67,373	4.5 (4.5-4.5)
Third or higher	100,156	19,722	5.0 (5.0-5.1)
Unknown	367,456	68,168	5.4 (5.4-5.4)
**Smoking status**			
Current smoker	201,192	34,045	5.9 (5.9-5.9)
Past smoker	403,203	82,292	4.9 (4.9-4.9)
Non-smoker	656,620	139,161	4.7 (4.7-4.7)
Unknown	166,695	44,190	3.8 (3.8-3.8)
**Year group**			
2006-2007	302,645	61,803	4.9 (4.9-4.9)
2008-2009	318,827	64,356	5.0 (4.9-5.0)
2010-2011	315,752	64,396	4.9 (4.9-4.9)
2012-2013	285,875	60,974	4.7 (4.7-4.7)
2014-2015	204,611	48,159	4.2 (4.2-4.3)

Abbreviations: CI – confidence interval

For peer review only - http://bmjopen.bmj.com/site/about/guidelines.xhtml

Figure captions

Figure 1: Flow diagram showing application of study inclusion and exclusion criteria

Figure 2: Women's consultation rate on each day in the first year following childbirth

For peer review only

For peer review only - http://bmjopen.bmj.com/site/about/guidelines.xhtml

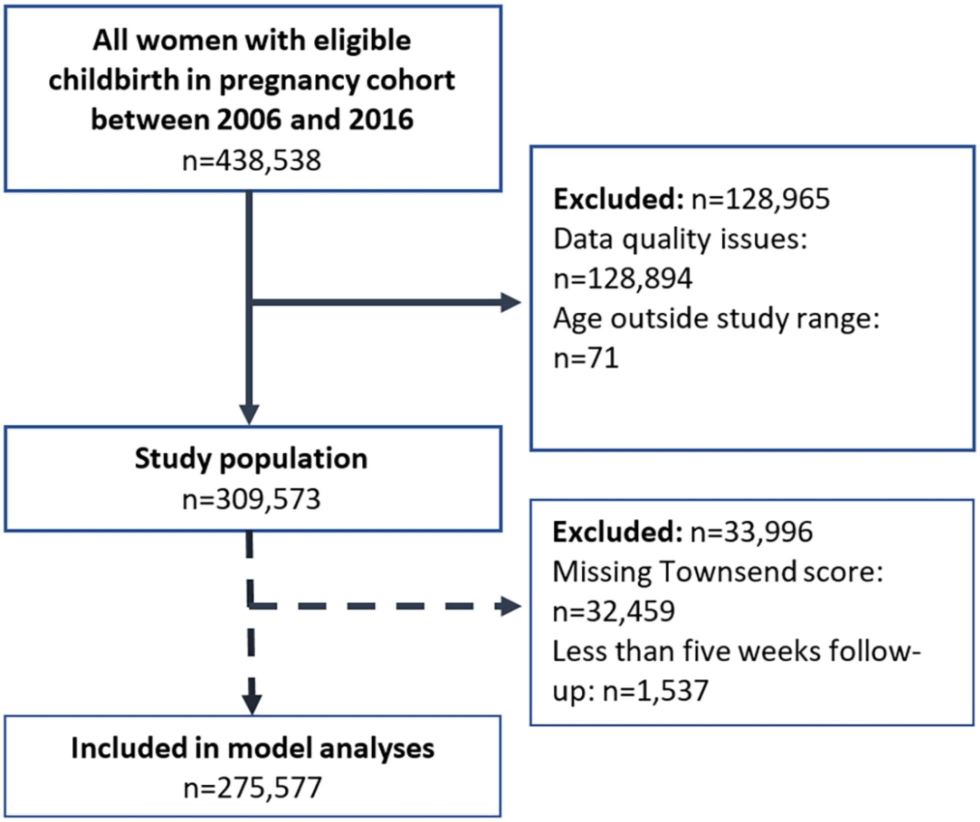

Figure 1: Flow diagram showing application of study inclusion and exclusion criteria

105x90mm (300 x 300 DPI)

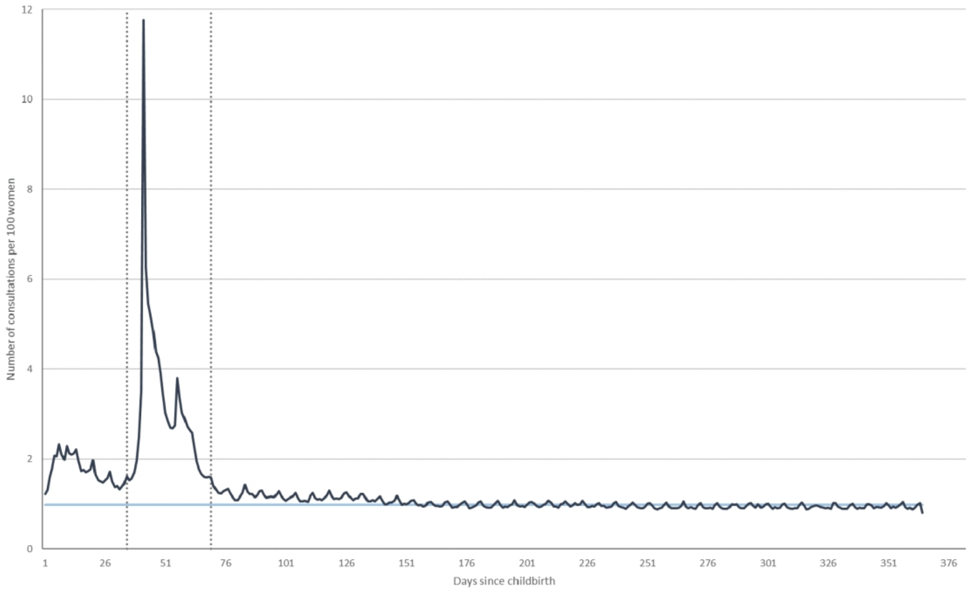

Figure 2: Women's consultation rate on each day in the first year following childbirth *Dotted lines indicate weeks 5 and 10, horizontal line indicates consultation rate after week 10.

148x90mm (300 x 300 DPI)

Holly Smith, 29/06/20
Postnatal_check_supplementary

Supplementary materials

Likelihood of having a postnatal check using a more sensitive outcome definition (any consultation between weeks 5-10)

Characteristic	All women n	Consultation in weeks 5-10 n (%)	Consultation in weeks 5-10: Unadjusted: IRR (95% CI)	Consultation in weeks 5-10: Age & deprivation adjusted: IRR (95% CI)
**Overall**	309,573	243,516 (78.7)		
**Maternal age (years)**				
15-19	9,568	6,977 (72.9)	0.94 (0.91-0.96)	0.94 (0.92-0.97)
20-24	43,116	32,429 (75.2)	0.96 (0.95-0.98)	0.97 (0.95-0.98)
25-29	77,698	60,597 (78.0)	0.99 (0.98-1.00)	0.99 (0.98-1.00)
30-34	98,269	78,671 (80.1)	1	1
35-39	64,171	51,504 (80.3)	1.00 (0.98-1.01)	1.00 (0.98-1.01)
40-44	15,908	12,674 (79.7)	0.99 (0.97-1.01)	0.99 (0.97-1.01)
45-49	843	664 (78.8)	0.98 (0.90-1.06)	0.97 (0.90-1.06)
**Townsend Score quintile**				
1-least deprived	58,583	48,142 (82.2)	1	1
2	53,656	43,336 (80.8)	1.00 (0.98-1.01)	1.00 (0.98-1.01)
3	62,023	49,169 (79.3)	0.99 (0.97-1.00)	0.99 (0.98-1.00)
4	58,506	45,574 (77.9)	0.98 (0.96-0.99)	0.98 (0.97-1.00)
5-most deprived	44,346	32,729 (73.8)	0.95 (0.93-0.96)	0.95 (0.94-0.97)
Missing	32,459	24,566 (75.7)	Excluded	Excluded
**Mode of delivery**				
Vaginal delivery	75,506	63,533 (86.8)	1	1
Caesarean	23,426	20,074 (85.7)	1.03 (1.01-1.04)	1.02 (1.01-1.04)
Unknown	210,641	159,533 (75.7)	0.96 (0.94-0.98)	0.96 (0.94-0.97)
**Parity**				
First	149,639	118,998 (79.5)	1	1
Second	69,355	53,969 (77.8)	0.97 (0.96-0.98)	0.97 (0.96-0.98)
Third or higher	20,113	15,258 (75.9)	0.95 (0.93-0.96)	0.94 (0.92-0.95)
Unknown	70,466	55,291 (78.5)	0.96 (0.95-0.97)	0.95 (0.94-0.96)
**Smoking status**				
Current smoker	34,634	27,236 (78.6)	0.99 (0.98-1.01)	1.01 (0.99-1.02)
Past smoker**	85,592	66,542 (77.7)	0.97 (0.96-0.98)	0.97 (0.96-0.98)
Non-smoker	143,349	115,019 (80.2)	1	1
Unknown	45,998	34,719 (75.5)	0.93 (0.92-0.94)	0.93 (0.92-0.94)
**Year group**				
2006-2007	63,793	50,496 (79.2)	1	1
2008-2009	66,319	52,571 (79.3)	1.00 (0.98-1.01)	1.00 (0.98-1.01)
2010-2011	66,478	52,819 (79.5)	1.00 (0.99-1.02)	1.01 (0.99-1.02)
2012-2013	63,180	49,788 (78.8)	1.00 (0.99-1.02)	1.00 (0.99-1.02)
2014-2015	49,803	37,842 (76.0)	0.97 (0.96-0.99)	0.97 (0.96-0.99)

* Abbreviations: IRR – incidence rate ratio, CI – confidence interval.
**Practice and woman are included as random effects terms in all models
***Models exclude women with missing Townsend score

For peer review only - http://bmjopen.bmj.com/site/about/guidelines.xhtml

Number of women who had a consultation in weeks 0-4 and/or weeks 5-10, % of cohort

Consultation in week 0-4 (down)/ Consultation in weeks 5-10 (across)	Yes	No	Total
Yes	89,605 (28.9%)	18,723 (6.0%)	108,328 (35.0%)
No	153,911 (49.7%)	47,334 (15.3%)	201,245 (65.0%)
Total	243,516 (78.7%)	66,057 (21.3%)	309,573

For peer review only

For peer review only - http://bmjopen.bmj.com/site/about/guidelines.xhtml

STROBE Statement—Checklist of items that should be included in reports of ***cohort studies***

	Item No	Recommendation	Page No
**Title and abstract**	1	(*a*) Indicate the study's design with a commonly used term in the title or the abstract<br>(*b*) Provide in the abstract an informative and balanced summary of what was done and what was found	1-2<br>2
**Introduction**			
Background/rationale	2	Explain the scientific background and rationale for the investigation being reported	4
Objectives	3	State specific objectives, including any prespecified hypotheses	4
**Methods**			
Study design	4	Present key elements of study design early in the paper	1-2
Setting	5	Describe the setting, locations, and relevant dates, including periods of recruitment, exposure, follow-up, and data collection	5-6
Participants	6	(*a*) Give the eligibility criteria, and the sources and methods of selection of participants. Describe methods of follow-up<br>(*b*) For matched studies, give matching criteria and number of exposed and unexposed	5-6
Variables	7	Clearly define all outcomes, exposures, predictors, potential confounders, and effect modifiers. Give diagnostic criteria, if applicable	6-7
Data sources/ measurement	8*	For each variable of interest, give sources of data and details of methods of assessment (measurement). Describe comparability of assessment methods if there is more than one group	5-7
Bias	9	Describe any efforts to address potential sources of bias	6
Study size	10	Explain how the study size was arrived at	5-8
Quantitative variables	11	Explain how quantitative variables were handled in the analyses. If applicable, describe which groupings were chosen and why	6-7
Statistical methods	12	(*a*) Describe all statistical methods, including those used to control for confounding<br>(*b*) Describe any methods used to examine subgroups and interactions<br>(*c*) Explain how missing data were addressed<br>(*d*) If applicable, explain how loss to follow-up was addressed<br>(*e*) Describe any sensitivity analyses	7<br>7<br>6-7<br>5-7<br>6-7
**Results**			
Participants	13*	(a) Report numbers of individuals at each stage of study—eg numbers potentially eligible, examined for eligibility, confirmed eligible, included in the study, completing follow-up, and analysed<br>(b) Give reasons for non-participation at each stage<br>(c) Consider use of a flow diagram	8<br>8<br>8, figure 1
Descriptive data	14*	(a) Give characteristics of study participants (eg demographic, clinical, social) and information on exposures and potential confounders<br>(b) Indicate number of participants with missing data for each variable of interest<br>(c) Summarise follow-up time (eg, average and total amount)	8<br>8, table1<br>8-9, table2
Outcome data	15*	Report numbers of outcome events or summary measures over time	8-9

For peer review only - http://bmjopen.bmj.com/site/about/guidelines.xhtml

Main results	16	(*a*) Give unadjusted estimates and, if applicable, confounder-adjusted estimates and their precision (eg, 95% confidence interval). Make clear which confounders were adjusted for and why they were included	8-9, all tables
		(*b*) Report category boundaries when continuous variables were categorized	6-7, tables
		(*c*) If relevant, consider translating estimates of relative risk into absolute risk for a meaningful time period	n/a
Other analyses	17	Report other analyses done—eg analyses of subgroups and interactions, and sensitivity analyses	8, supplementary material
**Discussion**			
Key results	18	Summarise key results with reference to study objectives	10
Limitations	19	Discuss limitations of the study, taking into account sources of potential bias or imprecision. Discuss both direction and magnitude of any potential bias	10
Interpretation	20	Give a cautious overall interpretation of results considering objectives, limitations, multiplicity of analyses, results from similar studies, and other relevant evidence	11-12
Generalisability	21	Discuss the generalisability (external validity) of the study results	11-12
**Other information**			
Funding	22	Give the source of funding and the role of the funders for the present study and, if applicable, for the original study on which the present article is based	13-14

*Give information separately for exposed and unexposed groups.

Note: An Explanation and Elaboration article discusses each checklist item and gives methodological background and published examples of transparent reporting. The STROBE checklist is best used in conjunction with this article (freely available on the Web sites of PLoS Medicine at http://www.plosmedicine.org/, Annals of Internal Medicine at http://www.annals.org/, and Epidemiology at http://www.epidem.com/). Information on the STROBE Initiative is available at http://www.strobe-statement.org.

For peer review only

BMJ Open

Postnatal checks and primary care consultations in the year following childbirth: an observational cohort study of 309,573 women in the UK, 2006-2016

Journal:	*BMJ Open*
Manuscript ID	bmjopen-2020-036835.R2
Article Type:	Original research
Date Submitted by the Author:	26-Aug-2020
Complete List of Authors:	Smith, Holly; University College London, Primary Care and Population Health<br>Saxena, Sonia; Imperial College London, School of Public Health;<br>Petersen, Irene; University College London Medical School, Department of Primary Care and Population health
**Primary Subject Heading**:	General practice / Family practice
Secondary Subject Heading:	Epidemiology, Obstetrics and gynaecology
Keywords:	Maternal medicine < OBSTETRICS, PRIMARY CARE, EPIDEMIOLOGY, REPRODUCTIVE MEDICINE

SCHOLARONE™
Manuscripts

I, the Submitting Author has the right to grant and does grant on behalf of all authors of the Work (as defined in the below author licence), an exclusive licence and/or a non-exclusive licence for contributions from authors who are: i) UK Crown employees; ii) where BMJ has agreed a CC-BY licence shall apply, and/or iii) in accordance with the terms applicable for US Federal Government officers or employees acting as part of their official duties; on a worldwide, perpetual, irrevocable, royalty-free basis to BMJ Publishing Group Ltd ("BMJ") its licensees and where the relevant Journal is co-owned by BMJ to the co-owners of the Journal, to publish the Work in this journal and any other BMJ products and to exploit all rights, as set out in our licence.

The Submitting Author accepts and understands that any supply made under these terms is made by BMJ to the Submitting Author unless you are acting as an employee on behalf of your employer or a postgraduate student of an affiliated institution which is paying any applicable article publishing charge ("APC") for Open Access articles. Where the Submitting Author wishes to make the Work available on an Open Access basis (and intends to pay the relevant APC), the terms of reuse of such Open Access shall be governed by a Creative Commons licence – details of these licences and which Creative Commons licence will apply to this Work are set out in our licence referred to above.

Other than as permitted in any relevant BMJ Author's Self Archiving Policies, I confirm this Work has not been accepted for publication elsewhere, is not being considered for publication elsewhere and does not duplicate material already published. I confirm all authors consent to publication of this Work and authorise the granting of this licence.

For peer review only

Holly Smith, 26/08/20
Postnatal_check_manuscript

Title:

Postnatal checks and primary care consultations in the year following childbirth: an observational cohort study of 309,573 women in the UK, 2006-2016

Authors:

Holly Christina Smith (0000-0001-6805-680X), Sonia Saxena (0000-0003-3787-2083), Irene Petersen (0000-0002-0037-7524).

Holly Christina Smith, PhD student, Department of Primary Care and Population Health, University College London, Royal Free Campus, Rowland Hill Street, London, NW3 2PF.

Sonia Saxena, Professor of Primary Care, School of Public Health, Faculty of Medicine, Imperial College London, 332 Reynolds Building, Charing Cross Campus, W6 8RP.

Irene Petersen, Professor of Epidemiology and Health Informatics, Department of Primary Care and Population Health, Institute of Epidemiology & Health, University College London, Royal Free Campus, Rowland Hill Street, London, NW3 2PF.

Correspondence to: Holly Christina Smith**:** Holly.dorning.18@ucl.ac.uk.

Word count:

3,581

For peer review only

Holly Smith, 26/08/20
Postnatal_check_manuscript

Abstract

Objective: To describe women's uptake of postnatal checks and primary care consultations in the year following childbirth.

Design: Observational cohort study using electronic health records.

Setting: UK primary care.

Participants: Women aged 16-49 years who had given birth to a single live infant recorded in The Health Improvement Network (THIN) primary care database in 2006-2016.

Main outcome measures: Postnatal checks and direct consultations in the year following childbirth.

Results: We examined 1,427,710 consultations in 309,573 women who gave birth to 241,662 children in 2006-2016. Of these women, 78.7% (243,516) had a consultation at the time of the postnatal check, but only 56.2% (174,061) had a structured postnatal check documented. Teenage women (aged 16 to 19 years) were 12% less likely to have a postnatal check compared with those aged 30-35 years (incidence rate ratio (IRR)=0.88, 95% CI: 0.85-0.91) and those living in the most deprived vs. least areas were 10% less likely (IRR=0.90, 95% CI: 0.88-0.92). Women consulted on average 4.8 times per women per year and 293,049 women (94.7%) had at least one direct consultation in the year after childbirth. Consultation rates were higher for those with a caesarean delivery (7.7 per women per year, 95% CI: 7.7-7.8). Consultation rates peaked during weeks 5-10 following birth (11.8 consultations/ 100 women) coinciding with the postnatal check.

Conclusions: Two in ten women did not have a consultation at the time of the postnatal and four in ten women have no record of receiving a structured postnatal check within the first ten weeks after giving birth. Teenagers and those from the most deprived areas are among the least likely to have a check. We estimate up to 350,400 women per year in the UK may be missing these opportunities for timely health promotion and to have important health needs identified following childbirth.

Keywords: Maternal Health, Postnatal Care, Primary Health Care, Electronic Health Records, Postpartum Period

For peer review only

Strengths and limitations of this study

- A major strength of this study is that it is among the largest population-based studies to date of postnatal care in the first 12 months.
- We drew on data from electronic health records which reflect real-world clinical practice in UK primary care.
- We are limited by what is recorded in a woman's electronic health record and genuine postnatal checks may have occurred in reality which were not accurately coded in primary care data.
- This study may underestimate the number of women who had a postnatal check compared with estimates based on survey data.

For peer review only

For peer review only - http://bmjopen.bmj.com/site/about/guidelines.xhtml

Introduction

Providing high quality comprehensive postnatal primary care is a global goal for improving maternal and child health in the first year of life [1]. In the United Kingdom (UK), nearly 800,000 women give birth each year [2] and every woman has access to midwives and health visitors for the first few days after delivery. They are then discharged to the care of their General Practitioner (GP) who invites them for a planned postnatal check 6-8 weeks after birth [3], as recommended by National Institute for Health and Care Excellence (NICE)[4] and the World Health Organisation (WHO) as part of routine postnatal care[5]. The maternal postnatal check provides a unique and timely opportunity for new mothers and health care professionals to evaluate their physical and mental health and assess how women are recovering after pregnancy and birth[6]. The postnatal check is also a point where women and primary care health professionals can discuss breastfeeding, postpartum contraception, smoking cessation, return to physical activity, and dietary advice, particularly after gestational weight gain. GPs also play a role in supporting parents to cope with managing day to day care and minor illness of infants and identify safeguarding concerns for new mothers and their babies.

The postnatal period is typically defined as the first six weeks after childbirth[7] and previous studies estimate 47% to 83% of women will report at least one health problem around eight weeks postpartum [8-10]. Historically, it was anticipated women would recover from pregnancy within this time [11]; however, there is increasing evidence that women have ongoing health needs throughout the first year [12] and even longer [13 14]. For example, up to 5% of women will require ongoing management of medical complications of gestational diabetes[15]; and others may need support with postpartum conditions such as postnatal depression which occurs in 1 in 6 [16]. Previous research may underestimate maternal morbidity as many women do not report symptoms or may be reluctant to seek support after childbirth [17].

From previous cross-sectional surveys from 1995 and 2010-2014, it would appear that up to 90% of women attend their postnatal checks, but selection bias of survey participants may overestimate this figure [18-20]. We found no contemporary studies showing patterns of primary care use for women following childbirth. The aim of this observational cohort study was to examine the prevalence of postnatal checks, explore factors associated with having a postnatal check and primary care consultations for women in the first year after childbirth.

For peer review only - http://bmjopen.bmj.com/site/about/guidelines.xhtml

Methods

UK healthcare

In the UK, healthcare is free at the point of delivery for all residents as part of the National Health Service (NHS). Primary care is typically the first point of contact and is largely delivered by GPs and other health care professionals (nurses and health visitors) within a practice. Information about patients and their health are collected during primary care consultations. This information is primarily used for clinical care but is also widely used for research through large healthcare databases.

Data source

We used one of the largest UK primary care databases, The Health Improvement Network (THIN) database. As of December 2016, THIN contained anonymised electronic health records for 16 million registered patients from 730 practices across the UK [21]. The database contains patient-level information on: demographics, prescribing, symptoms, procedures, prevention, lifestyle factors and diagnostics. Consultations can be linked to give a comprehensive picture of someone's care. Diagnostic and symptomatic information are categorised using Read codes, a hierarchical coding system [22]. Additional Health Data codes classify prevention and lifestyle information. Socioeconomic information is captured through Townsend score which provides a measure of material deprivation based on: where a person lives, unemployment, car ownership, home ownership and household overcrowding [23]. THIN is broadly representative of the UK population in terms of demographics, chronic disease and mortality; however, there is an over-representation of more affluent people [24].

Study population

A cohort of women who have given birth to a single live infant has previously been identified within THIN [25]. Births and date of childbirth were identified using a combination of an antenatal record, delivery record, postnatal care record, date of last menstrual period, or birth of a child matched to mother's record. This pre-existing cohort of women excluded more complex births, including multiple deliveries (twins, triplets etc.) and those with a known miscarriage, termination or stillbirth, so it is not possible to include these women in our study. This cohort contains information on approximately 650,000 pregnancies/childbirths in 1990-2016. In this study, we included women aged 15 to 49 years who gave birth between 1st January 2006 and 31st December 2015 from this cohort. Data quality criteria were also applied whereby practices which did not have acceptable computer use (ACU) or acceptable mortality rates (AMR) by the date of childbirth were excluded.

For peer review only - http://bmjopen.bmj.com/site/about/guidelines.xhtml

ACU is the date a practice was continuously entering on average at least two therapy records, one medical record and one additional health data record per patient per year [26]; and AMR is the date a practice has comparable mortality rates to the rest of the UK, given the size and demographics of the practice [27]. Women who had been registered at a practice for less than six months were also excluded. It was possible for women to have multiple childbirths in this study. Women were followed-up for 12 months to identify their primary use after each childbirth, censoring for maternal death or practice transfer.

Definition of variables

Postnatal check

We identified a postnatal check as any consultation at the time of the check (typically weeks 6-8) which had a specific Read code (beginning with '62R' or '62S') or Additional Health Data code ('1044100000' or '1044000000') identifying it as postnatal visit and/or check. Some women may receive this check slightly earlier or later, and on reviewing the data this window was expanded to weeks 5-10 after birth to include a peak in consultations. We identified substantial variation in the use of these codes by practices and change over time (data not shown). Therefore, we used a second and more sensitive approach where we assumed any consultation at the time of the routine appointment (weeks 5-10) was an opportunity for a postnatal check. The results of this second approach are included in supplementary materials.

Consultations

A primary care consultation was defined as any direct contact between a patient and a healthcare professional taking place: in practice, in a patient's home or by telephone. It was assumed only one consultation took place each day for each woman, therefore multiple records on the same date were grouped.

Patient and childbirth characteristics

We stratified our analysis by maternal age, number of births, Townsend score (described previously), smoking status, year (two-year bands) and mode of delivery. Women were assigned to a five-year bands according to their age. We used information in the mother's records as well as children registered within the same household at the time of birth to assign number of births (categorised as: First, Second, Third or higher, or Unknown). We used Townsend score quintiles whereby each woman is assigned to one of five groups of deprivation, from least to most deprived. We assigned women's smoking status as 'current smoker' (record of smoking at any time in the year after childbirth), 'past smoker' (record of smoking or being an ex-smoker in the two years prior to

For peer review only - http://bmjopen.bmj.com/site/about/guidelines.xhtml

childbirth and not a current smoker), ‘non-smoker’ or ‘unknown’. Mode of delivery was determined using the identifying pregnancy/childbirth Read codes and was broadly grouped into ‘caesarean’, ‘vaginal’ and ‘unknown’ based on classifications developed previously [25].

Statistical analysis

A table was derived to show characteristics of women at each childbirth. The crude consultation rate was calculated as the total number of consultations per total person-years, stratified by characteristics. To explore variation across the first year, consultation rate was calculated as the number of consultations on each day with number of women registered with a practice on that day as the denominator. Women who died or transferred practice were censored from the denominator each day. To examine those who had a postnatal check, firstly we calculated the crude proportion of women with the outcome in each patient strata. To explore variation by characteristic in more detail, we examined the likelihood of having a postnatal check between 5-10 weeks in women with at least five weeks follow-up and complete deprivation (Townsend score) information. We developed mixed-effects Poisson models to estimate how the likelihood of having a postnatal check between weeks 5-10 varied by maternal age, Townsend score, mode of delivery, number of births, smoking status and year. Three models were developed: unadjusted, age-adjusted and age-deprivation adjusted. Practice and woman (as women can have multiple childbirths) were included as random effects terms, and the log of follow-up time (between weeks 5-10) was included as an offset. All analyses were conducted using Stata V.16 (StataCorp, College Station, Texas, USA).

Patient and public involvement

Two public panels interested in primary care research provided feedback on the study outline. The groups were supportive of the idea to identify attendance of postnatal checks, they suggested we clearly identify groups who do not attend and to examine differences in a woman’s first vs subsequent childbirths. As a result, we have included additional analysis exploring attendance by patient characteristics and number of births.

For peer review only - http://bmjopen.bmj.com/site/about/guidelines.xhtml

Results

Participants

Between 1 January 2006 and 31 December 2016, 438,538 pregnancies/childbirths were identified in the pregnancy cohort within THIN data. Study inclusion and exclusion criteria were applied to these records which resulted in a final sample of 309,573 childbirths (Figure 1).

Characteristics of women

We identified 309,573 childbirths in this study related to 241,662 women. At childbirth, a third of the women were aged 30-34 years (31.7%). There were 21.1% in the least deprived Townsend quintile compared 16.0% in the most deprived (which is similar to the overall distribution in THIN [24]). Three quarters of women had a vaginal delivery and the rest had a caesarean birth (76.3% vs 23.7%). Of these, nearly half were a first birth (48%) and 22% were a second birth. Half of women were non-smokers (46.3%), compared to 11.2% being current smoker (Table 1).

Postnatal check

Overall, just over half of the women in our study (56%) had a structured postnatal check, i.e. 44% had no such records (Table 1). In this crude analysis, younger women and those from the most deprived areas were less likely to have a postnatal check (48% of those aged 15-19 years vs 59.5% of those aged 35-39 years; and 47.7% of those from the most deprived area vs 62.7% from the least).

After excluding those with less than five weeks of follow-up information, missing deprivation information, 275,577 women were included in additional analysis (Figure 1). Those aged 15-19 years were 12% less likely (incidence rate ratio (IRR)=0.88, 95% CI: 0.85-0.91) to have a postnatal check between weeks 5-10 relative to women aged 30-35 years (Table 2). Similarly, women from the most deprived areas were 10% less likely (IRR=0.90, 95% CI: 0.88-0.92) to have a postnatal check relative to those from the least deprived areas. Differences across other characteristics were less pronounced; and, adjusting for age, and age and deprivation, had little impact on differences across these other characteristics. The same trend across age and deprivation was identified when using a more sensitive approach to identify a potential postnatal check (any consultation between weeks 5-10), but with a higher proportion of women (78.7%) having a consultation (see supplementary material).

A small proportion of women in our study 18,723 (6.0%) had a consultation in the first four weeks but did not have a consultation in weeks 5-10 (see supplementary material). This compares to a

For peer review only - http://bmjopen.bmj.com/site/about/guidelines.xhtml

much greater proportion of women 89,605 (28.9%) who had both an early consultation and one in weeks 5-10.

Primary care consultations

Following the 309,573 childbirths, the majority (94.7%, n=293,049) of women had at least one direct consultation in the year after childbirth. A total of 1,427,710 direct consultations were identified, with women consulting on average 4.8 times/person-year (Table 3). The largest differences in consultation rate compared to the average is seen in those who had a caesarean delivery (7.7/ person-year, 95% CI: 7.7-7.8) and in current smokers (5.9/ person-year, 95% CI: 5.9-5.9). Consultation rate decreased over time, from 4.9/ person-year (95% CI: 4.9-4.9) in 2006-2007 to 4.2/ person-year in 2014-2015 (4.2-4.3). Consultation rates were broadly similar across other characteristics.

Across the first year the consultation rate was highest between weeks 5-10, with a peak of 11.8 consultations/ 100 women in week 6, coinciding with the postnatal check. Following this, the consultation rate fell to an average of 1 consultation/ 100 women (Figure 2).

For peer review only

Discussion

Main findings

We found that eight in 10 women had a consultation at the time of the postnatal check; however, only half of women had a record of receiving a structured postnatal check. Teenage women (aged 15-19 years) were 12% less likely to have a postnatal check compared with older women aged 30-35 years, and those living in the most deprived areas were 10% less likely compared to women from least deprived areas. Women consulted on average 4.8 times per year in the year after childbirth and 94.7% of women had at least one consultation in the year after childbirth. Those who had a caesarean delivery and smokers had higher than average consultation rates (7.7 times per women per year and 5.9 times per women per year respectively). Consultation rates decreased over time (from 4.9 time per woman per year in 2006-2007 to 4.2 times in 2014-2015). Across the first year, the consultation rate is highest in week six with a peak of 11.8 consultations/ 100 women, which coincides with the postnatal check, after week ten the consultation rate is flat with 1 consultation/ 100 women on each day.

Study strengths and weaknesses

This is among the largest (299,688 person years) population-based studies to date of postnatal care in the first 12 months. The use of electronic health records provides a reflection of real-world clinical practice allowing us to explore use of the postnatal check in a broad population (not reliant on patient participation). As with all studies of electronic health records however, we are limited by what has been recorded in a woman's record which could mean patient, birth and consultation characteristics may be missing or not accurate. We also recognise the limitations in using Read codes/AHD codes only to identify a postnatal check as there is variation in the use of these by general practice and genuine checks may not be coded as such in primary care data. To account for this, we repeated our analysis using a more sensitive definition of a postnatal check (any consultation in week 5-10). While we found a larger proportion (78.7%) having been in contact with primary care, the overall trends in terms of the sociodemographic information was consistent between the two approaches.

Findings in relation to previous studies

The characteristics of women in our cohort are broadly similar to all women who give birth in terms of age and mode of delivery in a comparison year of 2017[28,29]. However, our cohort has a greater proportion of women who live in less deprived areas compared to all births in England and Wales,

For peer review only - http://bmjopen.bmj.com/site/about/guidelines.xhtml

which limits the generalisability of our findings. We identified that those from more deprived areas were less likely to have a postnatal check which supports previous findings[20]. This may mean we overestimate the proportion of women who have a postnatal check compared to all women who give birth in the UK. Previous studies estimate that 85% to 91% of women in England have a postnatal check [18 19]; however only 56% of women in our study had evidence of having one. We would expect our estimates to be lower as we used electronic health records which capture a broader picture of real-world practice compared with previous studies that may have been subject to selection and recall bias. When using a more sensitive approach, where we considered any consultation in weeks 5-10 as evidence of a postnatal check, our findings are closer to those of previous studies, although we are aware that not all of these consultations would have covered topics meant for the postnatal check. It is likely the true number having a check lies between our two estimates. The consultation rate of 4.8 per person per year identified in our study is comparable to that found by others, when taking age, sex and reason for consultation into account[30].

There are several possible explanations for our findings of a low uptake of postnatal checks. It is possible that women do not want or feel they need advice from GPs; or invitations from the GP are not taken up either because women do not respond to them, or may find it difficult to access appointments. Alternatively a lack of recording in electronic health records may explain the apparently low rate of postnatal checks.

Implications of findings and future research recommendations

It is encouraging to find that the majority of women return to primary care at least once in the year after childbirth; however, it is concerning that four in ten women did not have a structured postnatal check documented and that consultation rates have declined over time. In UK, approximately 800,000 women give birth each year[2], our estimates (44%) then suggest that up to 350,400 women may be missing this key check. The postnatal period is a potentially vulnerable time for women and there could be serious consequences to not identifying women at risk of poor health or harm after childbirth[31]. The postnatal check has been shown to be a key contact to identify serious health needs such as postnatal depression, which affects 1 in 6 women after childbirth. [25] It also provides protected time and opportunities to improve women's health and wellbeing through preventative intervention such as timely access to contraception, advice about weight management or diet following gestational weight gain, or support to stop smoking can be given [4]. Our finding that younger women and those from more deprived areas are less likely to have a check is particularly important as they may be most likely to benefit. For example, contraceptive uptake is particularly

For peer review only - http://bmjopen.bmj.com/site/about/guidelines.xhtml

low in younger and more deprived groups[32], and offering timely access through the check could lead to fewer unwanted or repeat pregnancies for these women.

Our findings suggest practices may need to implement systems for follow up of women who have declined or missed a postnatal check. There is a need for better promotion of the benefits of attending the postnatal check at other times in the maternity pathway; such as during midwife or health visitor appointments, in hospital or birth units, or at other GP maternity and baby check-ups. Additionally, there are currently no known financial or quality-based incentives to document primary care activity in the postnatal period. This could lead to variation in services and underreporting of activity. It is vital to improve the documentation of this care to more accurately understand women's use of the postnatal check, more broadly their health needs and service use after childbirth, and ultimately improve care. This is particularly important as we identified that postnatal consultation rates declined over time. We recommend more research to explore the reasons behind the low uptake of postnatal checks and variation in consultation rates.

In this study our focus was on who had a postnatal check, and while NICE outlines the content of these appointments, few studies have explored what health needs are covered in actuality. This should be explored through further research to better understand what content and delivery are most effective for women and if attending a postnatal check leads to better outcomes. Current NICE guidance recommends the postnatal check take place 6 weeks after childbirth within primary care. There has been some evaluation of this timing and frequency,[33,34] and in particular how this relates to the early postnatal care women receive from midwives. Further high-quality studies are needed to determine the most effective timing of postnatal care consultations. It is also important to examine the accuracy of postnatal care in electronic health records and explore ways to improve this in future studies. Lastly, our study focused on the postnatal care use of women who had given birth to a single live infant only. Complex births, such as multiple deliveries or stillbirths are relatively rare (15.9 out of every 1,000 women giving birth in England and Wales had a multiple birth in 2016 and 4.4 per 1,000 births were a stillbirth)[35]. It is expected that these women would receive additional follow-up in specialist care and so would not represent the usual pathway back to primary care services. However, this has not been well investigated and future studies should explore if these women have different experiences of postnatal care. Furthermore, future studies could explore the differences in postnatal care use by ethnicity, country of birth, language spoken and refugee or asylum seeker status.

Conclusion

For peer review only - http://bmjopen.bmj.com/site/about/guidelines.xhtml

Two in ten women had no consultation at the time of the postnatal check and four in ten women have no record of receiving a structured postnatal check within the first ten weeks after giving birth, this is despite the majority of women returning to primary care at least once in the year after childbirth. Teenagers and those from the most deprived areas are among the least likely to have a check. We estimate up to 350,400 women per year in the UK may be missing these opportunities for timely health promotion and to have important health needs identified after childbirth.

For peer review only

For peer review only - http://bmjopen.bmj.com/site/about/guidelines.xhtml

Acknowledgements

We are very grateful to members of the public who generously gave their time to feedback on the outline of this study, in particular we are grateful to the many women who have shared their experiences of childbirth and beyond, and who serve as a continued source of inspiration for this research. We are also appreciative for the support of our funders.

Footnotes

Authors contributions

This study was designed and conceived by HCS, SS and IP. HCS conducted the data analysis and wrote the first draft of this manuscript. SS and IP made comments on the manuscript. HCS and IP had full access to the database and guarantee this manuscript is an honest, accurate, and transparent account of the study being reported; that no important aspects of the study have been omitted; and that any discrepancies from the study as planned have been explained.

Funding

This study is funded by the National Institute for Health Research (NIHR) School for Primary Care Research (SPCR) (project reference 549832). The views expressed are those of the authors and not necessarily those of the NIHR or the Department of Health and Social Care.

SS is funded by the National Institute for Health Research (NIHR) School for Public Health Research (SPHR) and NIHR Northwest London Applied Research Collaboration (ARC). The School for Public Health Imperial College London is also grateful for support from the Imperial NIHR Biomedical Research Centre.

The funders had no role in study design, data collection and analysis, decision to publish, or preparation of the manuscript.

Data sharing statement

No additional data are available.

Competing interests

All authors have completed the ICMJE uniform disclosure form at www.icmje.org/coi_disclosure.pdf and declare: HCS reports grants from National Institute of Health Research School for Primary Care Research and SS reports grants from NIHR School for Public Health Research, grants from NIHR NW

For peer review only - http://bmjopen.bmj.com/site/about/guidelines.xhtml

London Applied Research Collaboration, during the conduct of the study; grants from The Daily Mile Foundation Charity, outside the submitted work; no other relationships or activities that could appear to have influenced the submitted work.

Ethical approval and data access

Approval was received from the Scientific Review Committee on 10/04/2019 (THIN protocol number: 19THIN013). THIN is a registered trademark of Cegedim SA in the UK and other countries. Reference made to THIN database is intended to be descriptive of the data asset licensed by IQVIA. This work uses de-identified data provided by patients as a part of their routine primary care.

Copyright/license for publication

The Corresponding Author HCS has the right to grant on behalf of all authors and does grant on behalf of all authors, a worldwide licence to the Publishers and its licensees in perpetuity, in all forms, formats and media (whether known now or created in the future), to i) publish, reproduce, distribute, display and store the Contribution, ii) translate the Contribution into other languages, create adaptations, reprints, include within collections and create summaries, extracts and/or, abstracts of the Contribution, iii) create any other derivative work(s) based on the Contribution, iv) to exploit all subsidiary rights in the Contribution, v) the inclusion of electronic links from the Contribution to third party material where-ever it may be located; and, vi) licence any third party to do any or all of the above.

As our data are anonymised it is not possible to disseminate our findings to study participants.

For peer review only

References

1. United Nations. Transforming our world: The 2030 agenda for sustainable development, 2015.
2. Office for National Statistics. Births by mothers' usual area of residence in the UK [Data file]. Office for National Statistics, London, 2016. [cited 12 Dec 2019]. Avaliable from: https://www.ons.gov.uk/peoplepopulationandcommunity/birthsdeathsandmarriages/livebirths/datasets/birthsbyareaofusualresidenceofmotheruk.
3. NHS Choices. Your 6-week postnatal check [Available from: https://www.nhs.uk/conditions/pregnancy-and-baby/postnatal-check/ accessed 20/12/2018.
4. National Institute for Health and Care Excellence. Postnatal care up to 8 weeks after birth. NICE Clinical Guideline 37, 2006. https://www.nice.org.uk/guidance/cg37.
5. World Health Organisation, United Nations Population Fund, Unicef. Pregnancy, Childbirth, Postpartum and Newborn Care: A guide for essential practice. Third ed. Geneva, 2015.
6. Jakes AD, Oakeshott P, Bick D. The maternal six week postnatal check. *BMJ* 2019;367:l6482. doi: 10.1136/bmj.l6482 [published Online First: 2019/12/04]
7. World Health Organisation. WHO Technical Consultation on Postpartum and Postnatal Care, 2010.
8. Glazener CM, Abdalla M, Stroud P, et al. Postnatal maternal morbidity: extent, causes, prevention and treatment. *Br J Obstet Gynaecol* 1995;102(4):282.
9. Thompson JF, Roberts CL, Currie M, et al. Prevalence and persistance of health problems after childbirth: associations with parity and method of birth. *Birth* 2002;29(2):83-94.
10. MacArthur C, Lewis M, Knox EG. Health after childbirth. *Br J Obstet Gynaecol* 1991;98:1193-204.
11. Albers LL. Health problems after childbirth. *Journal of Midwifery & Women's Health* 2000;45(1):55-57.
12. Ansara D, Cohen MM, Gallop R, et al. Predictors of women's physical health problems after childbirth. *Journal of Psychosomatic Obstetrics & Gynecology* 2009;26(2):115-25. doi: 10.1080/01443610400023064
13. Dahlen HG. Urinary incontinence: where to now? *BJOG* 2016;123(6):1030. doi: 10.1111/1471-0528.13427 [published Online First: 2015/05/27]
14. MacArthur C, Wilson D, Herbison P, et al. Faecal incontinence persisting after childbirth: a 12 year longitudinal study. *BJOG* 2013;120(2):169-79. doi: 10.1111/1471-0528.12039 [published Online First: 2012/11/30]
15. National Institute for Health and Care Excellence. Diabetes in pregnancy: management from preconception to the postnatal period. NICE Guideline No. 3, 2015. https://www.nice.org.uk/guidance/ng3.
16. Shorey S, Chee CYI, Ng ED, et al. Prevalence and incidence of postpartum depression among healthy mothers: A systematic review and meta-analysis. *J Psychiatr Res* 2018;104:235-48. doi: 10.1016/j.jpsychires.2018.08.001 [published Online First: 2018/08/17]
17. Chou D, Tuncalp O, Firoz T, et al. Constructing maternal morbidity - towards a standard tool to measure and monitor maternal health beyond mortality. *BMC Pregnancy Childbirth* 2016;16:45. doi: 10.1186/s12884-015-0789-4 [published Online First: 2016/03/05]
18. Bick DE, MacArthur C. Attendance, content and relevance of the six week postnatal examination. *Midwifery* 1995(11):69-73.
19. Redshaw M, Heikkila K. Delivered with care: a national survey of women's experience of maternity care 2010: National Perinatal Epidemiology Unit, 2010.
20. Redshaw M, Henderson J. Safely delivered: a national survey of women's experiences of maternity care 2014: National Perinatal Epidemiology Unit, 2015.
21. Quintiles IMS. The Health Improvement Network Data Guide for Researchers, 2017.
22. Booth N. What are the Read Codes? *Health Libr Rev* 1994;11:177-82.

For peer review only - http://bmjopen.bmj.com/site/about/guidelines.xhtml

23. Townsend P, Phillimore P, Beattie A. Health and deprivation: inequality and the north. London: Routledge 1989.
24. Blak BT, Thompson M, Dattani H, et al. Generalisability of The Health Improvement Network (THIN) database: demographics, chronic disease prevalence and mortality rates. *Inform Prim Care* 2011;19:251-5.
25. Petersen I, McCrea RL, Sammon CJ, et al. Risks and benefits of psychotropic medication in pregnancy: cohort studies based on UK electronic primary care health records. *Health Technol Assess* 2016;20(23):1-176. doi: 10.3310/hta20230 [published Online First: 2016/04/01]
26. Horsfall L, Walters K, Petersen I. Identifying periods of acceptable computer usage in primary care research databases. *Pharmacoepidemiol Drug Saf* 2013;22(1):64-9. doi: 10.1002/pds.3368 [published Online First: 2012/11/06]
27. Maguire A, Blak BT, Thompson M. The importance of defining periods of complete mortality reporting for research using automated data from primary care. *Pharmacoepidemiol Drug Saf* 2009;18(1):76-83. doi: 10.1002/pds.1688 [published Online First: 2008/12/10]
28. Office for National Statistics. Births by Parents' Characteristics, England and Wales [data file]. https://www.ons.gov.uk/peoplepopulationandcommunity/birthsdeathsandmarriages/livebirths/datasets/birthsbyparentscharacteristics. Published 2017. Accessed June 18, 2020.
29. NHS Digital. NHS Maternity Statistics, 2017-18: Hospital Episode Statistics [data file]. http://digital.nhs.uk/pubs/maternity1718. Published 2018. Accessed June 18, 2020.
30. Wang Y, Hunt K, Nazareth I, et al. Do men consult less than women? An analysis of routinely collected UK general practice data. *BMJ Open* 2013;3(8) doi: 10.1136/bmjopen-2013-003320
31. Knight M, Bunch K, Tuffnell D, et al. Saving Lives, Improving Mothers' Care: Lessons learned to inform maternity care from the UK and Ireland Confidential Enquiries into Maternal Deaths and Morbidity 2014–16, 2018.
32. National Institute for Health and Care Excellence. Contraceptive services for under 25s. NICE Public health guideline No. 51, 2014. www.nice.org.uk/guidance/ph51.
33. Gunn J, Lumley J, Chondros P, Young D. Does an early postnatal check-up improve maternal health: Results from a randomised trial in Australian general practice. *BJOG An Int J Obstet Gynaecol*. 1998;105(9):991-997. doi:10.1111/j.1471-0528.1998.tb10263.x
34. MacArthur C, Winter HR, Bick DE, et al. Effects of redesigned community postnatal care on womens' health 4 months after birth: a cluster randomised controlled trial. *Lancet*. 2002;359(9304):378-385. doi:10.1016/S0140-6736(02)07596-7
35. Office for National Statistics. Birth Characteristics in England and Wales [data file]. https://www.ons.gov.uk/peoplepopulationandcommunity/birthsdeathsandmarriages/livebirths/datasets/birthcharacteristicsinenglandandwales. Published 2016. Accessed June 18, 2020.

For peer review only

For peer review only - http://bmjopen.bmj.com/site/about/guidelines.xhtml

Tables

Table 1: Characteristics of women who have given birth to a single live infant and proportion of those women with a structured postnatal check 5-10 weeks after childbirth

Characteristic	All women<br>n	Record of postnatal check in weeks 5-10<br>n (% across the row)
**Overall**	309,573	174,061 (56.2)
**Maternal age (years)**		
15-19	9,568	4,599 (48.1)
20-24	43,116	21,763 (50.5)
25-29	77,698	42,417 (54.6)
30-34	98,269	57,308 (58.3)
35-39	64,171	38,154 (59.5)
40-44	15,908	9,347 (58.8)
45-49	843	473 (56.1)
**Townsend Score quintile**		
1-least deprived	58,583	36,752 (62.7)
2	53,656	32,326 (60.3)
3	62,023	35,413 (57.1)
4	58,506	31,601 (54.0)
5-most deprived	44,346	21,138 (47.7)
Missing	32,459	16,831 (51.9)
**Mode of delivery**		
Vaginal delivery	75,506	46,634 (61.8)
Caesarean	23,426	14,384 (61.4)
Unknown	210,641	113,043 (53.7)
**Number of births**		
First	149,639	84,010 (56.1)
Second	69,355	39,269 (56.6)
Third or higher	20,113	10,781 (53.6)
Unknown	70,466	40,001 (56.8)
**Smoking status**		
Current smoker	34,634	18,199 (52.6)
Past smoker	85,592	47,464 (55.5)
Non-smoker	143,349	82,420 (57.5)
Unknown	45,998	25,978 (56.5)
**Year group**		
2006-2007	63,793	36,863 (57.8)
2008-2009	66,319	38,124 (57.5)
2010-2011	66,478	37,897 (57.0)
2012-2013	63,180	34,896 (55.2)
2014-2015	49,803	26,281 (52.8)

For peer review only - http://bmjopen.bmj.com/site/about/guidelines.xhtml

Holly Smith, 26/08/20
Postnatal_check_manuscript

Table 2: Mixed-effects Poisson estimates of the likelihood of having a postnatal check for women who had given birth to a single live infant by age, Townsend score, mode of delivery, number of births, smoking status and year group; unadjusted, and adjusted for age and deprivation

Characteristic	n (%)	Record of postnatal check in weeks 5-10: Unadjusted: IRR (95% CI)	Age & deprivation adjusted: IRR (95% CI)
**Overall**	275,577		
**Maternal age (years)**			
15-19	8,704 (3.2)	0.88 (0.85-0.91)	0.89 (0.87-0.92)
20-24	38,503 (14.0)	0.92 (0.91-0.94)	0.93 (0.92-0.95)
25-29	68,751 (25.0)	0.97 (0.96-0.98)	0.97 (0.96-0.99)
30-34	86,889 (31.5)	1	1
35-39	57,533 (20.9)	1.00 (0.99-1.02)	1.00 (0.99-1.01)
40-44	14,428 (5.2)	0.99 (0.97-1.01)	0.99 (0.97-1.01)
45-49	769 (0.3)	0.96 (0.87-1.05)	0.96 (0.87-1.05)
**Townsend Score quintile**			
1-least deprived	58,304 (21.2)	1	1
2	53,370 (19.4)	0.99 (0.98-1.01)	1.00 (0.98-1.01)
3	61,681 (22.4)	0.97 (0.95-0.98)	0.97 (0.96-0.99)
4	58,165 (21.1)	0.95 (0.93-0.97)	0.96 (0.95-0.98)
5-most deprived	44,057 (16.0)	0.90 (0.88-0.92)	0.92 (0.90-0.93)
**Mode of delivery**			
Vaginal delivery	68,202 (76.6)	1	1
Caesarean	20,828 (23.4)	1.02 (1.00-1.04)	1.01 (0.99-1.03)
Unknown	186,547	0.99 (0.97-1.01)	0.99 (0.97-1.01)
**Number of births**			
First	132,164 (48.0)	1	1
Second	62,535 (22.7)	0.99 (0.98-1.01)	0.98 (0.97-0.99)
Third or higher	18,504 (6.7)	0.95 (0.93-0.97)	0.94 (0.92-0.96)
Unknown	62,374 (22.6)	0.96 (0.95-0.97)	0.95 (0.94-0.96)
**Smoking status**			
Current smoker	31,494 (11.4)	0.94 (0.92-0.95)	0.96 (0.95-0.98)
Past smoker	76,941 (27.9)	0.96 (0.95-0.97)	0.96 (0.95-0.98)
Non-smoker	126,497 (45.9)	1	1
Unknown	40,645 (14.8)	0.95 (0.94-0.97)	0.95 (0.93-0.96)
**Year group**			
2006-2007	58,606 (21.3)	1	1
2008-2009	60,212 (21.9)	0.99 (0.98-1.01)	0.99 (0.98-1.01)
2010-2011	59,183 (21.5)	1.00 (0.99-1.02)	1.00 (0.99-1.02)
2012-2013	55,099 (20.0)	0.99 (0.97-1.00)	0.99 (0.97-1.01)
2014-2015	42,477 (15.4)	0.95 (0.94-0.97)	0.95 (0.94-0.97)

Abbreviations: IRR – incidence rate ratio, CI – confidence interval.
Practice and woman are included as random effects terms in all models
Models exclude women with less than five weeks of follow-up information and missing Townsend score

For peer review only - http://bmjopen.bmj.com/site/about/guidelines.xhtml

Table 3: Crude consultation rate in the first year after childbirth of women who had given birth to a single live infant per/ person-year, by characteristic

Characteristic	Number of consultations	Person-years	Rate of consultations per person-year (95% CI)
**Overall**	1,427,710	299,688	4.8 (4.8-4.8)
**Maternal age (years)**			
15-19	46,087	9,135	5.0 (5.0-5.1)
20-24	211,905	41,212	5.1 (5.1-5.2)
25-29	371,051	74,994	4.9 (4.9-5.0)
30-34	437,873	95,385	4.6 (4.6-4.6)
35-39	283,912	62,581	4.5 (4.5-4.6)
40-44	72,941	15,561	4.7 (4.7-4.7)
45-49	3,941	821	4.8 (4.7-5.0)
**Townsend Score quintile**			
1-least deprived	269,502	57,128	4.7 (4.7-4.7)
2	242,798	52,137	4.6 (4.6-4.7)
3	285,632	59,972	4.8 (4.7-4.8)
4	274,005	56,420	4.9 (4.8-4.9)
5-most deprived	212,965	42,609	5.0 (5.0-5.0)
Missing	142,808	31,422	4.5 (4.5-4.6)
**Mode of delivery**			
Vaginal delivery	511,769	73,251	7.0 (7.0-7.0)
Caesarean	176,199	22,743	7.7 (7.7-7.8)
Unknown	739,742	203,694	3.6 (3.6-3.6)
**Number of births**			
First	655,616	144,425	4.5 (4.5-4.6)
Second	304,482	67,373	4.5 (4.5-4.5)
Third or higher	100,156	19,722	5.0 (5.0-5.1)
Unknown	367,456	68,168	5.4 (5.4-5.4)
**Smoking status**			
Current smoker	201,192	34,045	5.9 (5.9-5.9)
Past smoker	403,203	82,292	4.9 (4.9-4.9)
Non-smoker	656,620	139,161	4.7 (4.7-4.7)
Unknown	166,695	44,190	3.8 (3.8-3.8)
**Year group**			
2006-2007	302,645	61,803	4.9 (4.9-4.9)
2008-2009	318,827	64,356	5.0 (4.9-5.0)
2010-2011	315,752	64,396	4.9 (4.9-4.9)
2012-2013	285,875	60,974	4.7 (4.7-4.7)
2014-2015	204,611	48,159	4.2 (4.2-4.3)

Abbreviations: CI – confidence interval

For peer review only - http://bmjopen.bmj.com/site/about/guidelines.xhtml

Figure captions

Figure 1: Flow diagram showing application of study inclusion and exclusion criteria

Figure 2: Women's consultation rate on each day in the first year following childbirth

For peer review only

For peer review only - http://bmjopen.bmj.com/site/about/guidelines.xhtml

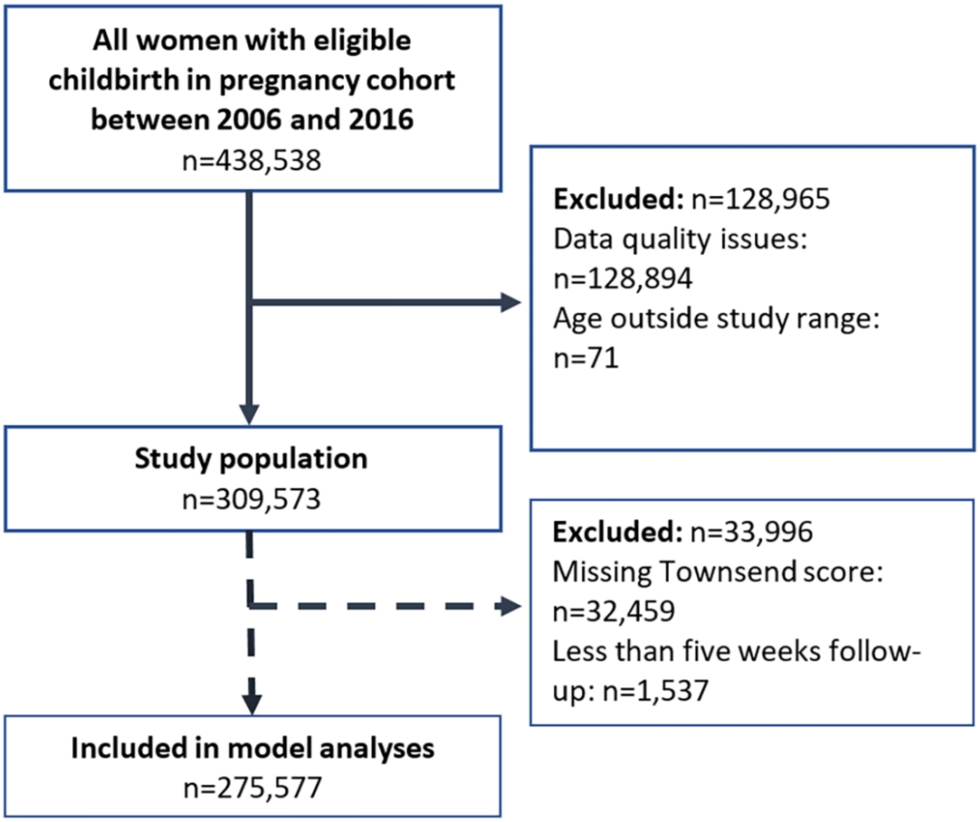

Figure 1: Flow diagram showing application of study inclusion and exclusion criteria

105x90mm (300 x 300 DPI)

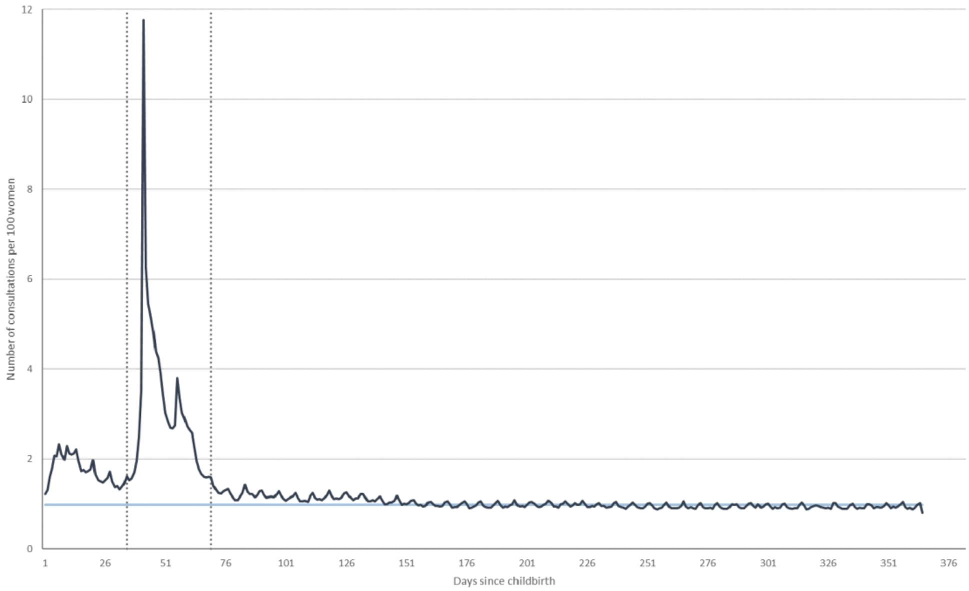

Figure 2: Women's consultation rate on each day in the first year following childbirth *Dotted lines indicate weeks 5 and 10, horizontal line indicates consultation rate after week 10.

148x90mm (300 x 300 DPI)

Holly Smith, 29/06/20
Postnatal_check_supplementary

Supplementary materials

Likelihood of having a postnatal check using a more sensitive outcome definition (any consultation between weeks 5-10)

Characteristic	All women n	Consultation in weeks 5-10 n (%)	Consultation in weeks 5-10: Unadjusted: IRR (95% CI)	Consultation in weeks 5-10: Age & deprivation adjusted: IRR (95% CI)
**Overall**	309,573	243,516 (78.7)		
**Maternal age (years)**				
15-19	9,568	6,977 (72.9)	0.94 (0.91-0.96)	0.94 (0.92-0.97)
20-24	43,116	32,429 (75.2)	0.96 (0.95-0.98)	0.97 (0.95-0.98)
25-29	77,698	60,597 (78.0)	0.99 (0.98-1.00)	0.99 (0.98-1.00)
30-34	98,269	78,671 (80.1)	1	1
35-39	64,171	51,504 (80.3)	1.00 (0.98-1.01)	1.00 (0.98-1.01)
40-44	15,908	12,674 (79.7)	0.99 (0.97-1.01)	0.99 (0.97-1.01)
45-49	843	664 (78.8)	0.98 (0.90-1.06)	0.97 (0.90-1.06)
**Townsend Score quintile**				
1-least deprived	58,583	48,142 (82.2)	1	1
2	53,656	43,336 (80.8)	1.00 (0.98-1.01)	1.00 (0.98-1.01)
3	62,023	49,169 (79.3)	0.99 (0.97-1.00)	0.99 (0.98-1.00)
4	58,506	45,574 (77.9)	0.98 (0.96-0.99)	0.98 (0.97-1.00)
5-most deprived	44,346	32,729 (73.8)	0.95 (0.93-0.96)	0.95 (0.94-0.97)
Missing	32,459	24,566 (75.7)	Excluded	Excluded
**Mode of delivery**				
Vaginal delivery	75,506	63,533 (86.8)	1	1
Caesarean	23,426	20,074 (85.7)	1.03 (1.01-1.04)	1.02 (1.01-1.04)
Unknown	210,641	159,533 (75.7)	0.96 (0.94-0.98)	0.96 (0.94-0.97)
**Parity**				
First	149,639	118,998 (79.5)	1	1
Second	69,355	53,969 (77.8)	0.97 (0.96-0.98)	0.97 (0.96-0.98)
Third or higher	20,113	15,258 (75.9)	0.95 (0.93-0.96)	0.94 (0.92-0.95)
Unknown	70,466	55,291 (78.5)	0.96 (0.95-0.97)	0.95 (0.94-0.96)
**Smoking status**				
Current smoker	34,634	27,236 (78.6)	0.99 (0.98-1.01)	1.01 (0.99-1.02)
Past smoker**	85,592	66,542 (77.7)	0.97 (0.96-0.98)	0.97 (0.96-0.98)
Non-smoker	143,349	115,019 (80.2)	1	1
Unknown	45,998	34,719 (75.5)	0.93 (0.92-0.94)	0.93 (0.92-0.94)
**Year group**				
2006-2007	63,793	50,496 (79.2)	1	1
2008-2009	66,319	52,571 (79.3)	1.00 (0.98-1.01)	1.00 (0.98-1.01)
2010-2011	66,478	52,819 (79.5)	1.00 (0.99-1.02)	1.01 (0.99-1.02)
2012-2013	63,180	49,788 (78.8)	1.00 (0.99-1.02)	1.00 (0.99-1.02)
2014-2015	49,803	37,842 (76.0)	0.97 (0.96-0.99)	0.97 (0.96-0.99)

* Abbreviations: IRR – incidence rate ratio, CI – confidence interval.
**Practice and woman are included as random effects terms in all models
***Models exclude women with missing Townsend score

For peer review only - http://bmjopen.bmj.com/site/about/guidelines.xhtml

Number of women who had a consultation in weeks 0-4 and/or weeks 5-10, % of cohort

Consultation in week 0-4 (down)/ Consultation in weeks 5-10 (across)	Yes	No	Total
Yes	89,605 (28.9%)	18,723 (6.0%)	108,328 (35.0%)
No	153,911 (49.7%)	47,334 (15.3%)	201,245 (65.0%)
Total	243,516 (78.7%)	66,057 (21.3%)	309,573

For peer review only

For peer review only - http://bmjopen.bmj.com/site/about/guidelines.xhtml

STROBE Statement—Checklist of items that should be included in reports of ***cohort studies***

	Item No	Recommendation	Page No
**Title and abstract**	1	(*a*) Indicate the study's design with a commonly used term in the title or the abstract<br>(*b*) Provide in the abstract an informative and balanced summary of what was done and what was found	1-2<br>2
**Introduction**			
Background/rationale	2	Explain the scientific background and rationale for the investigation being reported	4
Objectives	3	State specific objectives, including any prespecified hypotheses	4
**Methods**			
Study design	4	Present key elements of study design early in the paper	1-2
Setting	5	Describe the setting, locations, and relevant dates, including periods of recruitment, exposure, follow-up, and data collection	5-6
Participants	6	(*a*) Give the eligibility criteria, and the sources and methods of selection of participants. Describe methods of follow-up<br>(*b*) For matched studies, give matching criteria and number of exposed and unexposed	5-6
Variables	7	Clearly define all outcomes, exposures, predictors, potential confounders, and effect modifiers. Give diagnostic criteria, if applicable	6-7
Data sources/ measurement	8*	For each variable of interest, give sources of data and details of methods of assessment (measurement). Describe comparability of assessment methods if there is more than one group	5-7
Bias	9	Describe any efforts to address potential sources of bias	6
Study size	10	Explain how the study size was arrived at	5-8
Quantitative variables	11	Explain how quantitative variables were handled in the analyses. If applicable, describe which groupings were chosen and why	6-7
Statistical methods	12	(*a*) Describe all statistical methods, including those used to control for confounding<br>(*b*) Describe any methods used to examine subgroups and interactions<br>(*c*) Explain how missing data were addressed<br>(*d*) If applicable, explain how loss to follow-up was addressed<br>(*e*) Describe any sensitivity analyses	7<br>7<br>6-7<br>5-7<br>6-7
**Results**			
Participants	13*	(a) Report numbers of individuals at each stage of study—eg numbers potentially eligible, examined for eligibility, confirmed eligible, included in the study, completing follow-up, and analysed<br>(b) Give reasons for non-participation at each stage<br>(c) Consider use of a flow diagram	8<br>8<br>8, figure 1
Descriptive data	14*	(a) Give characteristics of study participants (eg demographic, clinical, social) and information on exposures and potential confounders<br>(b) Indicate number of participants with missing data for each variable of interest<br>(c) Summarise follow-up time (eg, average and total amount)	8<br>8, table1<br>8-9, table2
Outcome data	15*	Report numbers of outcome events or summary measures over time	8-9

For peer review only

Main results	16	(*a*) Give unadjusted estimates and, if applicable, confounder-adjusted estimates and their precision (eg, 95% confidence interval). Make clear which confounders were adjusted for and why they were included	8-9, all tables
		(*b*) Report category boundaries when continuous variables were categorized	6-7, tables
		(*c*) If relevant, consider translating estimates of relative risk into absolute risk for a meaningful time period	n/a
Other analyses	17	Report other analyses done—eg analyses of subgroups and interactions, and sensitivity analyses	8, supplementary material
**Discussion**			
Key results	18	Summarise key results with reference to study objectives	10
Limitations	19	Discuss limitations of the study, taking into account sources of potential bias or imprecision. Discuss both direction and magnitude of any potential bias	10
Interpretation	20	Give a cautious overall interpretation of results considering objectives, limitations, multiplicity of analyses, results from similar studies, and other relevant evidence	11-12
Generalisability	21	Discuss the generalisability (external validity) of the study results	11-12
**Other information**			
Funding	22	Give the source of funding and the role of the funders for the present study and, if applicable, for the original study on which the present article is based	13-14

*Give information separately for exposed and unexposed groups.

Note: An Explanation and Elaboration article discusses each checklist item and gives methodological background and published examples of transparent reporting. The STROBE checklist is best used in conjunction with this article (freely available on the Web sites of PLoS Medicine at http://www.plosmedicine.org/, Annals of Internal Medicine at http://www.annals.org/, and Epidemiology at http://www.epidem.com/). Information on the STROBE Initiative is available at http://www.strobe-statement.org.

For peer review only
